# Epigenetic Reprogramming Potentiates ICAM1 Antibody Drug Conjugates in Preclinical Models of Melanoma

**DOI:** 10.1002/advs.202400203

**Published:** 2024-06-14

**Authors:** Peng Zhang, Changjuan Tao, Ye Lu, Peijing Li, Xing Wang, Yujie Dai, Yun Xi, Takaya Shimura, Xinfang Li, Jianmin Fang, Liu Yang, Dawei He, Peng Guo

**Affiliations:** ^1^ Department of Medical Oncology Zhejiang Provincial People's Hospital Hangzhou Zhejiang 310022 China; ^2^ Key Laboratory of Tumor Molecular Diagnosis and Individualized Medicine of Zhejiang Province Zhejiang Provincial People's Hospital People's Hospital of Hangzhou Medical College Hangzhou Zhejiang 310014 China; ^3^ Hangzhou Institute of Medicine (HIM) Chinese Academy of Sciences Hangzhou Zhejiang 310022 China; ^4^ Department of Radiation Oncology The Cancer Hospital of the University of Chinese Academy of Sciences Zhejiang Cancer Hospital Hangzhou Zhejiang 310022 China; ^5^ Key Laboratory of Head and Neck Cancer Translational Research of Zhejiang Province Zhejiang Cancer Hospital Hangzhou Zhejiang 310022 China; ^6^ Department of Head and Neck Surgery The Cancer Hospital of the University of Chinese Academy of Sciences Zhejiang Cancer Hospital Hangzhou Zhejiang 310022 China; ^7^ MabPlex International Yantai Shandong 264006 China; ^8^ Department of Pathology The Cancer Hospital of the University of Chinese Academy of Sciences Zhejiang Cancer Hospital Hangzhou Zhejiang 310022 China; ^9^ Department of Gastroenterology and Metabolism Nagoya City University Graduate School of Medical Sciences Nagoya 467–8601 Japan; ^10^ School of Materials Science and Engineering Tianjin University Tianjin 300072 China; ^11^ Department of Urology Children's Hospital of Chongqing Medical University Chongqing China

**Keywords:** antibody drug conjugate, decitabine, ICAM1, melanoma, targeted therapy

## Abstract

Therapeutic benefits and underlying biomechanism(s) of antibody drug conjugates (ADC) in combination with other targeted therapeutics are largely unknown. Here, the synergy between ADC and epigenetic drug decitabine (DAC), a clinically approved DNA methylation inhibitor, in multiple preclinical models of melanoma specifically investigated. Mechanistically, the underlying biomechanisms of how DAC cooperatively worked with ICAM1 antibody conjugated DNA topoisomerase I inhibitor DXd (I1‐DXd) is elucidated. DAC treatment significantly enhanced anti‐tumor efficacy of I1‐DXd by upregulating antigen expression, enhancing antibody internalization and potentiating tumor sensitivity by epigenetically reprogramming of melanoma. Meanwhile, I1‐DXd/DAC combination also exerted regulatory effects on tumor microenvironment (TME) by enhancing tumor infiltration of innate and adaptive immune cells and improving penetration of ADCs with a boosted antitumor immunity. This study provides a rational ADC combination strategy for solid tumor treatment.

## Graphical abstract

Zhang et al. identify that ICAM1‐ADC exerted potent antitumor activity in melanoma. Further, decitabine could enhance antitumor efficacy of ICAM1‐ADC with multiple mechanisms, mainly including synergistically boosting anti‐tumor immunity; upregulating ICAM1 expression on melanoma cell; enhancing tumor penetration of ICAM1‐ADC. These findings provide rationale for ICAM1‐ADC as candidate for melanoma and pursue DAC as a combination partner for ICAM1‐ADCs.

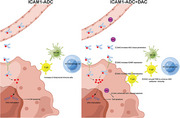

## 1 Introduction

Melanoma is the most aggressive and deadly type of skin cancers, accounting for over 80% of skin cancer deaths despite representing only 1% of skin cancers.^[^ ^1^ ^]^ Patients with advanced or metastatic melanoma have limited treatment options after tumor progression on immune‐checkpoint therapy and targeted therapy including v‐RAF murine sarcoma viral oncogene homolog B1 (BRAF)/mitogen‐activated protein kinase (MEK) inhibitors. A large retrospective cohort study from 5 international melanoma centers reported that the median overall survival of advanced melanoma patients after resistance to BRAF inhibitors was only 2.9 months,^[^ ^2^ ^]^ highlighting an urgent and unmet medical need for more effective targeted therapeutics.

Antibody‐drug conjugates (ADCs) are emerging therapeutic agents demonstrating excellent efficacy against various refractory and metastatic solid tumors.^[^ ^3^ ^]^ ADC could deliver highly cytotoxic payloads specifically to target‐expressing cancer cells by covalently conjugating the payloads with a tumor‐homing monoclonal antibody. Previous attempts to develop anti‐melanoma ADCs included DEDN6526A targeting endothelin B receptor (ETBR) ^[^ ^4^ ^]^ and glembatumumab vedotin targeting transmembrane glycoprotein NMB (GPNMB).^[^ ^5^ ^]^ The two aforementioned ADCs, both carried antimitotic payload monomethyl auristatin E (MMAE), were not progressed beyond phase I and phase II trials, due to limited efficacy and dose‐limiting toxicity. Therefore, there is still an urgent need to optimize ADC for melanoma by utilizing different targets and cytotoxic payloads. In our previous work, intercellular adhesion molecule 1(ICAM1) was identified as a promising cell membrane target for melanoma through an unbiased and quantitative screening.^[^ ^6^ ^]^ ICAM1 is a type I membrane‐bound glycoprotein, belonging to the immunoglobulin (Ig) superfamily.^[^ ^7^ ^]^ Moreover, high ICAM1 expression also correlates with poor prognosis in melanoma and drug resistance including BRAF/MEK inhibitors.^[^ ^6^, ^8^ ^]^ Our previous studies reported that ICAM1 ADCs are potent in ablating multiple refractory solid tumors including triple‐negative breast cancer ^[^ ^9^ ^]^ and anaplastic thyroid cancer.^[^ ^10^ ^]^ Here we evaluated two proof‐of‐principle ICAM1 ADCs with different mechanisms of action (MoA) for melanoma treatment in vitro and in vivo.

More importantly, to achieve more durable responses and higher response, combination therapies of ADCs and other anticancer drugs hold the promise of providing synergistic or additive benefits against refractory tumors via potentiating ADC treatment.^[^ ^11^ ^]^ A promising opportunity is targeting non‐mutational epigenetic tumor microenvironment, a hallmark of cancer with important roles in cancer development and progression.^[^ ^12^ ^]^ To address this, we performed an unbiased screening of melanoma cells with a library of 29 validated chemical probes that selectively target different epigenetic regulators. According to the screening results, we identified the DNA methylation (DNMT) inhibitor decitabine (DAC, an FDA‐approved and specific hypomethylating agent by inactivation of DMNTs) as the potential partner drug for ADC combination therapy. In the latter settings of this study, we explored the synergistic effects of ICAM1‐ADC/DAC combination therapy in multiple preclinical models of melanoma. Furthermore, we elucidated the key biological mechanisms that how DAC can work in synergy with ICAM1‐ADC by remodeling tumor microenvironments (TME).

Taken together, this study queried the single‐agent efficacy of two different ICAM1‐ADCs in preclinical models of melanoma. In addition, to unlock the potential of ADC for cancer therapy, we also sought to develop a strategy to enhance the efficacy of ICAM1‐ADC through combination with DAC, although the efficacy of ICAM1‐ADC as a single agent was remarkable across the preclinical models.

## 2 Results

### 2.1 Potency of ICAM1‐ADC monotherapy for melanoma

**Figure 1:**
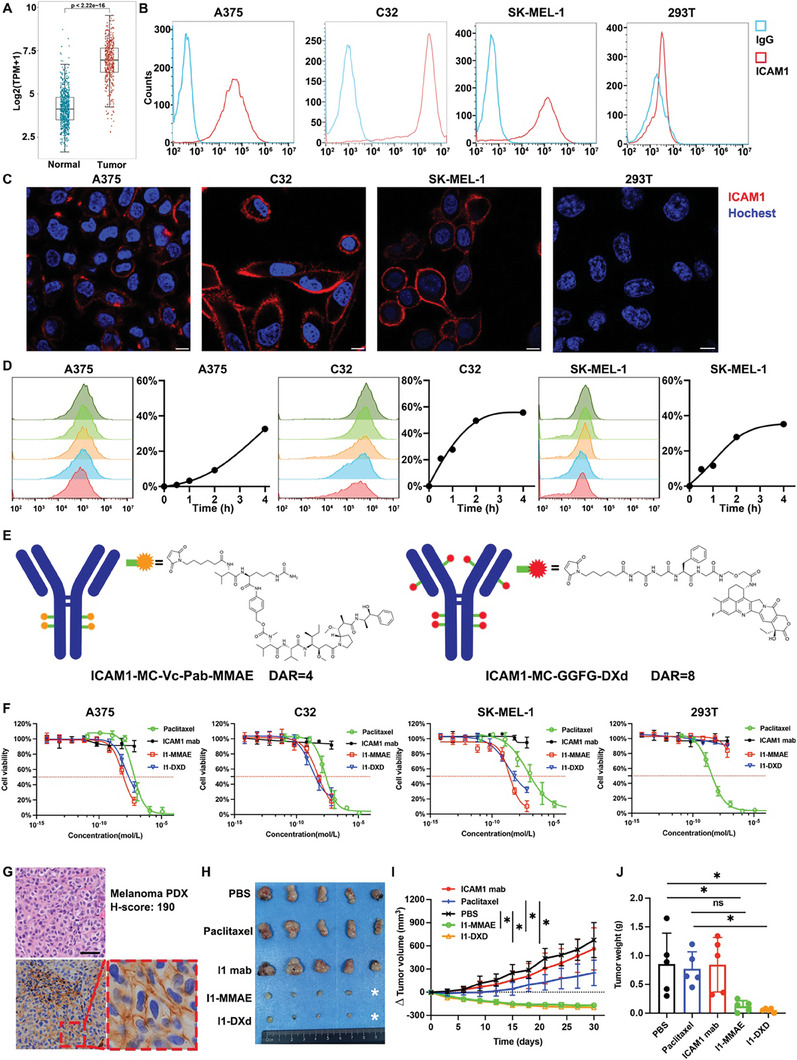
ICAM1‐ADCs show potent anti‐tumor efficacy in melanoma as monotherapy. (A) Box plots compare ICAM1 mRNA levels via melanoma to normal tissue; (B) Human melanoma cell and normal 293T surface expression of ICAM1 by flow cytometry (PE‐labeled antibody); (C) Representative images of IF staining of ICAM1 in human melanoma cells and normal 293T cell. Scale bar 10 µm; (D) Internalization curve of ICAM1 in melanoma cells (A375, C32 and SK‐MEL‐1) quantified by flow cytometry; (E) Schematic illustration and chemical structures of two ADC linkers and payloads; (F) In vitro cell growth inhibitory activity in A375, C32, SK‐MEL‐1 and 293T cell. Each point represents the mean and SD (n = 3). The red horizontal dotted line indicates the half maximal inhibitory; (G) Representative images of HE and IHC staining for ICAM1 in melanoma PDX model. Scale bar 50 µm; (H) Image of excised subcutaneous PDX tumors from mice treated with PBS (sham), I1 mab, I1‐MMAE, or I1‐DXd group (n = 5 per group); (I) Δ tumor volume in PDX model by tumor volume measurement by caliper. *p < 0.05; **p < 0.01; Bonferroni‐adjusted p value < 0.05 was considered statistically significant. (J) Tumor mass at the endpoint of PDX tumors quantified by weight. *p < 0.05; **p < 0.01; Bonferroni‐adjusted p value < 0.05 was considered statistically significant.

We have previously identified ICAM1 as a potential target for melanoma.^[^ ^6^ ^]^ The overexpression of ICAM1 in melanoma was subsequently clinically validated by the Cancer Genome Atlas Program (TCGA) and Human Protein Atlas (HPA) datasets. The expression of ICAM1 in TCGA melanoma patients was significantly high than normal controls (t = ‐39.681; *p* < 0.001; **Figure** **1**A). In HPA datasets, ICAM1 IHC staining intensity in 9/12 patients was scored strong and 3/12 patients were scored moderate (Supplement Figure S1A). In TCGA melanoma cohort, the prognosis of high‐ICAM1 expression group was significantly worse than low‐ICAM1 expression group (low‐ICAM1 group vs. high ICAM1 group: hazard ratio 0.61, p < 0.001; log‐rank p < 0.001; Supplement Figure S1B). There was a positive correlation between the mRNA levels of ICAM1 and the presence of BRAF mutations, which are among the most common genetic alterations in melanoma (r = 0.23, p < 0.001; Supplement Figure S1C). Further, the cell line mRNA expression matrix of melanoma, which was obtained from the Cancer Dependency Map dataset (https://depmap.org/portal/), also validated the overexpression of ICAM1 in most of 81 melanoma cell lines (Supplement Figure S1D). Then, three established melanoma cell lines with different ICAM1 expression (C32, high ICAM1 expression, ranking 3/81; SK‐MEL‐1, medium ICAM1 expression, ranking 41/81; A375, medium‐low ICAM1 expression, ranking 67/81) and normal 293T cell line as the ICAM1‐negative control were used to perform in vitro experiments. The overexpression of ICAM1 in melanoma was identified with immunofluorescent (IF) staining and flow cytometry and ICAM1 was predominantly localized on cytoplasmic membranes of melanoma cell lines (Figure 1B‐C and Supplement Figure S1E). In comparison, ICAM1 expression of 293T was undetectable (Figure 1B‐C and Supplement Figure S1E). The internalization of ICAM1 antibody was also evaluated with two independent approaches: time‐dependent flow cytometry and IF staining. The internalization rate of ICAM1 antibody at 4 h post‐incubation was determined as 32.6% (A375), 55.6% (C32) and 35.2% (SK‐MEL‐1), respectively (Figure 1D). The IF staining images intuitively validated that PE‐conjugated ICAM1 antibodies initially bound on cytoplasmic membrane of melanoma cells and then were gradually internalized with incubation time (Supplement Figure S1F). These results strongly support that ICAM1 is a potential drug delivery target of ADCs for melanoma.

To evaluate the efficacy of ADCs with different MoAs for melanoma, we evaluated two in‐house ICAM1‐ADCs (namely I1‐MMAE and I1‐DXd) previously developed by us ^[^ ^10^ ^]^ via conjugating monoclonal ICAM1 antibodies with two clinically proven linker and payload combinations: GGFG‐DXd and MC‐Vc‐Pab‐MMAE (Figure 1E). The DARs of I1‐DXd and I1‐MMAE were determined by HIC as 8 and 4, respectively (Supplement Figure S1G‐H), in consistency with clinical ADCs with the same linker and payload combination.^[^ ^3^, ^13^ ^]^ I1‐DXd acts on melanoma cells via its DNA topoisomerase I inhibitor DXd payload whereas I1‐MMAE through microtubule inhibitor MMAE payload. As shown in Figure 1F, both I1‐DXd and I1‐MMAE show potent in vitro efficacies against all three melanoma cell lines with IC50 values of 7.7 and 11.7nM for A375; 1.6 and 5.2nM for C32; 2.1 and 2.6nM for SK‐MEL‐1, respectively. It is noteworthy that the IC50s of IC1‐MMAE and I1‐DXd were both lower than paclitaxel, which is currently recommended to treat melanoma by NCCN guideline. In comparison, no cytotoxicity was observed in normal 239T cells due to the lack of ICAM1 expression (Figure 1F).

To evaluate the in vivo efficacy of ICAM1‐ADCs, we first established a melanoma patient‐derived xenograft (PDX) model (Figure 1G and Supplement Figure S2A). The HE staining of PDX faithfully retains the morphology of original tumors and the H score of ICAM1 expression in PDX was 190 (Figure 1G). In this PDX model, treatment with I1‐DXd or I1‐MMAE was initiated at the intravenous (i.v.) dosage of 5 mg/kg/week via tail vein injection. As controls, tumor‐bearing mice were treated with PBS, ICAM1 antibody alone or paclitaxel as controls at same dosage and time interval. Analysis via two‐way ANOVA demonstrated significant differences in drug efficacy across various groups (F = 30.43, p < 0.001). Subsequent post hoc testing with Bonferroni adjustments indicated that both I1‐DXd and I1‐MMAE treatments markedly reduced tumor growth compared to the PBS and paclitaxel controls at the study endpoint (day 30). Specifically, comparisons showed significant differences in tumor volume reduction between the PBS and I1‐DXd groups (Bonferroni‐adjusted p = 0.010), PBS and I1‐MMAE groups (Bonferroni‐adjusted p = 0.010), paclitaxel and I1‐DXd (Bonferroni‐adjusted p = 0.035) as well as the paclitaxel and I1‐MMAE groups (Bonferroni‐adjusted p = 0.041), as illustrated in Figures 1H and 1I. Notably, there was no significant difference in tumor volume reduction between the I1‐DXd and I1‐MMAE groups in the melanoma PDX model (Bonferroni‐adjusted p > 0.05).

Correlatively, the terminal tumor weights were determined as 0.83±0.56g (PBS), 0.84±0.47g (ICAM1 mab), 0.61±0.19g (paclitaxel), 0.16±0.07g (I1‐MMAE) and 0.08±0.02g (I1‐DXd), respectively. The one‐way ANOVA results showed the tumor mass weight at end point among different groups was statistically different (F = 6.695, p = 0.001). The post hoc with Bonferroni analysis revealed that the terminal tumor weights in I1‐DXd and I1‐MMAE groups were significantly lower than PBS (PBS vs. I1‐DXd, Bonferroni‐adjusted p = 0.017; PBS vs. I1‐MMAE, Bonferroni‐adjusted p = 0.034). The tumor mass weight at end point in I1‐DXd group was significantly lower than paclitaxel group (Paclitaxel vs. I1‐DXd, Bonferroni‐adjusted p = 0.041), while the difference between I1‐MMAE and paclitaxel group was not statistically different (Paclitaxel vs. I1‐MMAE, Bonferroni‐adjusted p = 0.082), as is shown in Figure 1J. No obvious body weight changes were observed in tested groups (Supplement Figure S2B), suggesting that ICAM1‐ADCs were well tolerated without off‐target toxicity in mice. These in vivo results support that ICAM1‐ADC monotherapy has potent anti‐melanoma activity for melanoma.

### 2.2 Synergistic anti‐tumor efficacy of ICAM1‐ADCs in combination with DAC in vitro and in vivo

**Figure 2:**
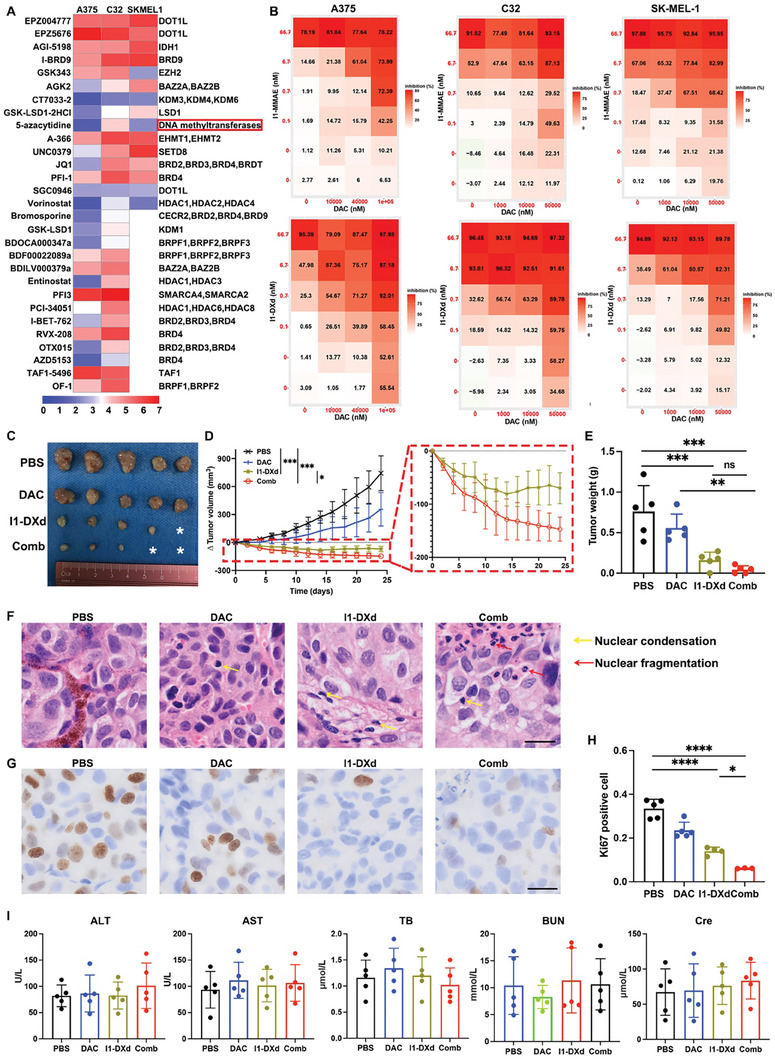
Combination with DAC significantly enhances the ICAM1‐ADC anti‐tumor efficacy in vitro and in vivo. (A) Chemical screen of 29 epigenetic probes identifies DNMT1 inhibitor as potential therapeutic target for melanoma. The color scale represents the z‐score of IC50. The lower IC 50 values indicated stronger anti‐proliferative activities effect in melanoma cells; (B) Combined inhibitory effects of various combination matrix of ICAM1‐ADCs (0‐66.7 nM) and DAC (A375: 0–100 µM; C32 and SK‐MEL‐1:0‐50 µM) in melanoma cells; (C) Image of excised subcutaneous PDX tumor from mice treated with PBS (sham), DAC, I1‐DXd, or Combo group (n = 5 per group); (D) Δ tumor volume curve in PDX model by tumor volume measurement by caliper. *p < 0.05; ***p < 0.001; Bonferroni‐adjusted p value < 0.05 was considered statistically significant. (E) Tumor mass weight at end point of subcutaneous PDX tumors. **p < 0.01; ***p < 0.001; Bonferroni‐adjusted p value < 0.05 was considered statistically significant. (F) Representative HE image of tumor in different groups. Scale bar 20µm. Yellow arrow indicated nuclear condensation; red arrow indicated nuclear fragmentation; (G) Representative Ki67 IHC staining image of tumor in different groups. Scale bar 20µm; (H) Quantitation of Ki67 positive cell proportion in different groups. *p < 0.05; ***p < 0.001; Bonferroni‐adjusted p value < 0.05 was considered statistically significant. (I) Blood chemistry parameters (ALT, AST, TB, BUN and Cre) measured when treated mice were sacrificed in different groups.

The abnormal epigenetic mechanism gives rise to oncogenic properties and is a hallmark of melanoma.^[^ ^14^ ^]^ To identify a potent epigenetic inhibitor as a partner for ADC combination therapy, we first performed a cell‐based chemical screening by 29 epigenetic inhibitor candidates from Genomics of Drug Sensitivity in Cancer (GDSC2) for melanoma cell growth inhibition. The 29 epigenetic chemical compounds, which are drug candidates currently in preclinical or clinical development, were key epigenetic regulatory proteins by Structural Genomics Consortium (SGC) collection and classical epigenetic targets.^[^ ^15^ ^]^ As is shown in **Figure** **2**A, DNMTs inhibitor, KDMs inhibitor, DOT1L inhibitor and HDACs inhibitor showed consistently low IC50s across all three melanoma cell lines among 29 epigenetic chemical compounds. In selecting a partner drug for ADC, we considered both IC50 values and clinical potential. Among the four candidates, KDMs inhibitor and DOT1L inhibitor lack clinical data. While HDACs inhibitor has been trialed as monotherapy, its efficacy in combination therapy remains unexplored. In contrast, DAC has undergone clinical evaluation in combination with temozolomide for melanoma treatment,^[^ ^16^ ^]^ demonstrating promising outcomes with a disease control rate (DCR) of 61% and a median overall survival (OS) of 12.4 months in a cohort of 35 patients (2 complete responses [CR], 4 partial responses [PR], and 14 stable diseases [SD]). This performance suggests a potential advantage over the historical 1‐year OS rate. The observed clinical tolerance and synergistic efficacy indicated DAC as a preferable partner for ADC combination therapy. Besides, analysis of Cancer Dependency Map dataset ^[^ ^17^ ^]^ also validated DNMT1 knockout by CRISPR/Cas9 gives high essential gene score in the epigenetic targets across melanoma cell lines, featuring a critical role for DNMT1 in melanoma (Supplement Figure S2C). We validated the DNMT1 overexpression in melanoma by TCGA dataset (Supplement Figure S2D). Previous studied demonstrated that DNMT1 was a hallmark of melanoma and indicating poorer survival in melanoma patients.^[^ ^14^, ^18^ ^]^ In TCGA of melanoma cohort, melanoma patients with high DNMT1 expression exhibited poorer disease‐free survival, but the difference was not statistically significant (Log‐rank p = 0.083; Supplement Figure S2E). Given decitabine (DAC), a clinically approved DNMT inhibitor, has shown promising efficacy and clinical safety in phase I clinical trials for melanoma,^[^ ^16^, ^19^ ^]^ DAC was selected to combine with ICAM1‐ADCs to explore the potential synergistic antitumor efficacy in vitro and in vivo. In the in vitro experiments, tumor cell inhibition of each DAC/ADC combination was presented with SynergyFinder (a web application for interactive analysis and visualization of multi‐drug combination response data) to probe combination effects in a dose‐response matrix. The results showed that a higher dosage of ADC caused even higher inhibition of cell growth rate in combination with the corresponding DAC treatment (Figure 2B). To quantify the synergistic effect of different ADCs (I1‐DXd and I1‐MMAE) and DAC, synergy scores were calculated using the highest single agent (HSA) model method by SynergyFinder.^[^ ^20^ ^]^ The HSA synergy scores of I1‐DXd and DAC combination were determined as 20.1 for A375; 11.0 for C32 and 14.4 for SK‐MEL‐1 (Supplement Figure S2F), respectively. The HSA synergy scores I1‐MMAE and DAC combination were determined as 19.2 for A375; 6.3 for C32 and 10.3 for SK‐MEL‐1, respectively (synergy scores less than ‐10 indicated that the interaction between two drugs is likely to be antagonistic; synergy scores from ‐10 to 10 indicated that the interaction between two drugs is likely to be additive; synergy scores larger than 10 indicated that the interaction between two drugs is likely to be synergistic). The combination of I1‐DXd and DAC in A375 (medium‐low ICAM1 expression) cells achieved the highest synergy score. Based on the results that synergy scores of I1‐DXd and DAC combination are consistently higher than 10, this finding indicates that the interaction of two drugs is likely to be synergistic, warranting further investigation in vivo.

We next evaluated the synergistic effects of I1‐DXd and DAC combination (Combo) in the previously established PDX melanoma model. Two‐way ANOVA analysis indicated significant differences in drug efficacy across groups (F = 39.15, p < 0.001). Subsequent Bonferroni‐adjusted post hoc tests showed that the combination therapy group significantly reduced tumor growth compared to both PBS and I1‐DXd groups at the study endpoint (day 24) (PBS vs. Combo, Bonferroni‐adjusted p = 0.002; I1‐DXd vs. Combo, Bonferroni‐adjusted p = 0.011), as depicted in Figures 2C‐D. One‐way ANOVA revealed significant variations in terminal tumor weights among the groups (F = 15.58, p < 0.001), with Bonferroni‐adjusted post hoc analysis showing significantly lower tumor weights in the I1‐DXd and combination therapy groups compared to PBS (PBS vs. I1‐DXd, Bonferroni‐adjusted p = 0.001; PBS vs. Combo, Bonferroni‐adjusted p < 0.001). While the average tumor weight at the endpoint was lower in the combination therapy group than in the I1‐DXd group, this difference was not statistically significant (0.044g vs. 0.160g, Bonferroni‐adjusted p > 0.05), as illustrated in Figure 2E. Meanwhile, two‐way ANOVA analysis indicated that no obvious weight loss was observed among the treatment groups (F = 0.096, p = 0.961) (Supplement Figure S2G), suggesting that the combination of I1‐DXd and DAC was well tolerated in mice. Moreover, pathological staining reveals that Combo group exhibited more apoptotic phenotypes (nuclear condensation and nuclear fragmentation; Figure 2F). Ki67 immunohistochemistry was employed to evaluate cell proliferation, as illustrated in Figures 2G‐H. One‐way ANOVA analysis identified significant differences in Ki67 positivity across groups (F = 53.01, p < 0.001). Bonferroni‐adjusted post hoc comparisons indicated significantly reduced Ki67 positive cells in both the I1‐DXd and Combo groups compared to the PBS control (PBS vs. I1‐DXd, Bonferroni‐adjusted p < 0.001; PBS vs. Combo, Bonferroni‐adjusted p < 0.001). Notably, the Combo group exhibited a further significant reduction in Ki67 positive cells compared to the I1‐DXd group (Bonferroni‐adjusted p = 0.043), indicating enhanced anti‐proliferative effects. Additionally, one‐way ANOVA analysis showed no significant differences in liver function tests (ALT, AST), total bilirubin (TB), creatinine, and blood urea nitrogen (BUN) across the groups (ALT: F = 0.388, p = 0.764; AST: F = 0.255, p = 0.856; TB: F = 0.696, p = 0.568; Creatinine: F = 0.273, p = 0.843; BUN: F = 0.385, p = 0.765), as shown in Figure 2I. This indicates that the treatments did not induce significant hepatotoxicity or nephrotoxicity, maintaining a favorable safety profile. Besides, no evidence of physiological changes and organ‐related damages was observed in H&E staining of major normal organs (Supplement Figure S2H), indicating that combination of I1‐DXd and DAC is biologically safe without off‐target toxicities in the mouse model.

**Figure 3:**
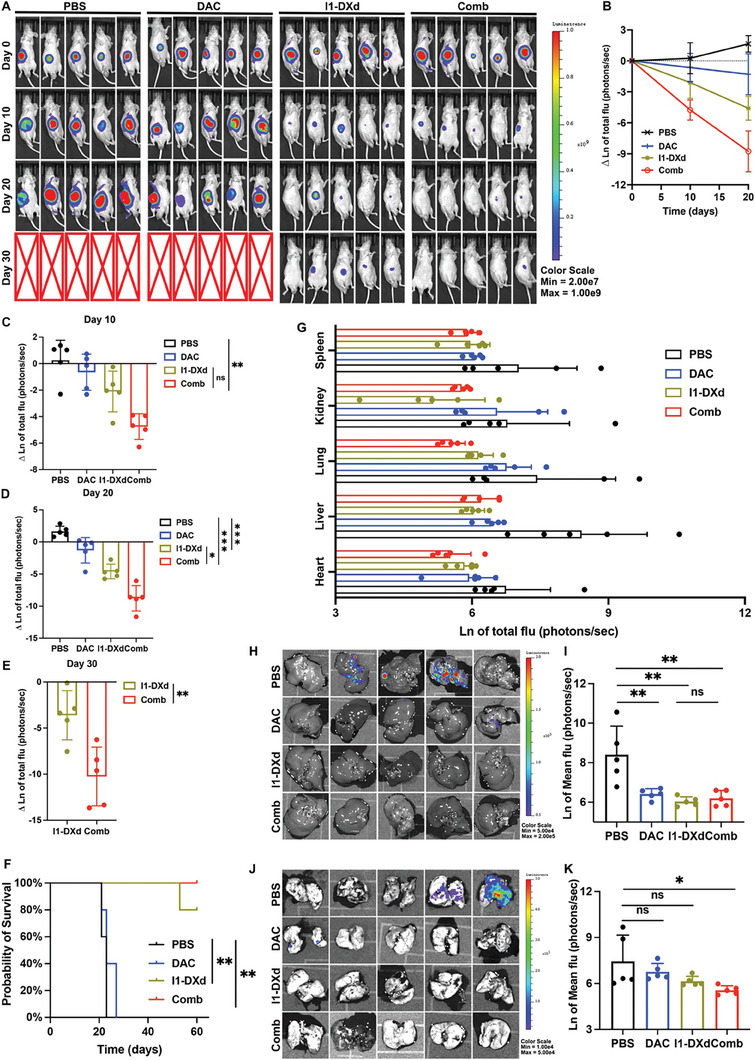
I1‐DXd monotherapy or in combination with DAC inhibits melanoma metastasis and improves survival in mice bearing A375‐luc xenograft tumors. (A) Representative bioluminescent images of mice A375‐luc xenograft tumors in different groups at different time points after injection; (B) Bioluminescence signal intensity at different time points in different groups (n = 5 per group); (C) Quantitative analysis of natural logarithm of tumor bioluminescence signal intensity in different groups at day 10. **p < 0.01; Bonferroni‐adjusted p value < 0.05 was considered statistically significant. (D) Quantitative analysis of natural logarithm of tumor bioluminescence signal intensity in different groups at day 20. **p < 0.01; ***p < 0.001; Bonferroni‐adjusted p value < 0.05 was considered statistically significant. (E) Quantitative analysis of natural logarithm of tumor bioluminescence signal intensity in different groups at day 30. **p < 0.01. (F) Kaplan–Meier survival curve of mice in different groups; (G) Bioluminescence signal intensity of major organs (heart, liver, lung, kidney and spleen) when mice were sacrificed in different groups; (H) Representative bioluminescence images of liver in different groups; (I) Quantitative analysis of liver metastasis burden as depicted from natural logarithm of bioluminescence signal intensity. **p < 0.01; Bonferroni‐adjusted p value < 0.05 was considered statistically significant. (J) Representative bioluminescence images of lung in different groups; (K) Quantitative analysis of lung metastasis burden as depicted from natural logarithm of bioluminescence signal intensity. *p < 0.05; Bonferroni‐adjusted p value < 0.05 was considered statistically significant.

We next chose luciferase‐transfected A375 cells (A375‐luc), which has moderate‐low expression of ICAM1, to construct an orthotopic xenograft tumor model to further confirm the anti‐tumor and anti‐metastatic efficacy of I1‐DXd monotherapy or combined with DAC. In **Figure** **3**A‐B, Combo group facilitated the maximal reduction of tumoral bioluminescence intensity among all tested groups. The napierian logarithm (ln) of tumor bioluminescence intensity across different groups was analyzed using two‐way ANOVA. This analysis demonstrated significant differences in drug effects (F = 39.04, p < 0.0001). On day 10, the combination therapy group showed a reduction in fluorescence intensity compared to the I1‐DXd group, although this difference did not reach statistical significance (I1‐DXd vs. Combo, Bonferroni‐adjusted p = 0.087), as depicted in Figure 3C. By day 20, Bonferroni‐adjusted post hoc analysis indicated that the combination therapy group significantly reduced fluorescence intensity relative to both the PBS and I1‐DXd groups (PBS vs. Combo, Bonferroni‐adjusted p < 0.001; I1‐DXd vs. Combo, Bonferroni‐adjusted p = 0.034), shown in Figure 3D. On day 30, fluorescence intensity in the combination therapy group was significantly lower than in the I1‐DXd group (t = 3.573, p = 0.007), as illustrated in Figure 3E. Correlatively, Kaplan‐Meier survival analysis indicated that the combination therapy group demonstrated a significantly improved survival benefit over the PBS group (PBS vs. I1‐DXd, p = 0.003; PBS vs. Combo, p = 0.003; Figure 3F). Additionally, one‐way ANOVA was utilized to assess differences in survival times among groups, revealing significant disparities (F = 478.5, p < 0.001). Bonferroni‐adjusted post hoc analysis further confirmed that both the I1‐DXd and the combination groups had significantly extended survival times compared to the PBS group (PBS vs. I1‐DXd, Bonferroni‐adjusted p < 0.001; PBS vs. Combo, Bonferroni‐adjusted p < 0.001), as illustrated in Supplement Figure S2I. However, there was no significant difference in survival times between the I1‐DXd and combination therapy groups (Bonferroni‐adjusted p > 0.05). The major organs were also excised for examining spontaneous metastases of A375‐luc tumors when tumor‐bearing mice were sacrificed. The bioluminescence intensity of major organs in different groups was compared by one‐way ANOVA analysis. The one‐way ANOVA analysis revealed that the bioluminescence intensity in kidney (F = 2.526, p = 0.094) and spleen (F = 2.984, p = 0.062) among different groups were not statistically different (Figure 3G). The bioluminescence intensity in heart (F = 3.374, p = 0.044), liver (F = 10.36, p = 0.001) and lung (F = 3.775, p = 0.032) among different groups were statistically different. The post hoc with Bonferroni analysis of heart bioluminescence intensity revealed that Combo group could significantly attenuate fluorescence intensity in comparison with PBS group (PBS vs. Combo, Bonferroni‐adjusted p = 0.046). The fluorescence intensity difference in heart between PBS and I1‐DXd group was not statistically different (PBS vs. I1‐Dxd, Bonferroni‐adjusted p = 0.220). The post hoc with Bonferroni analysis of bioluminescence intensity in liver revealed that I1‐DXd and Combo group could both significantly attenuate fluorescence intensity in comparison with PBS group (PBS vs. I1‐Dxd, Bonferroni‐adjusted p = 0.001; PBS vs. Combo, Bonferroni‐adjusted p = 0.002), as shown in Figure 3H‐I. The post hoc with Bonferroni analysis of bioluminescence intensity in lung revealed that Combo group could significantly attenuate fluorescence intensity in comparison with PBS group (PBS vs. Combo, Bonferroni‐adjusted p = 0.034). The fluorescence intensity difference in lung between PBS and I1‐DXd group was not statistically different (PBS vs. I1‐Dxd, Bonferroni‐adjusted p = 0.257), as shown in Figure 3J‐K. Specially, Combo group could effectively attenuate melanoma metastasis to lung and liver tissues. These findings indicate that DAC may work in synergy with ADCs to attenuate melanoma primary tumor growth and its metastases.

### 2.3 I1‐DXd monotherapy boosts anti‐tumor immunity and synergistically enhanced by DAC

**Figure 4:**
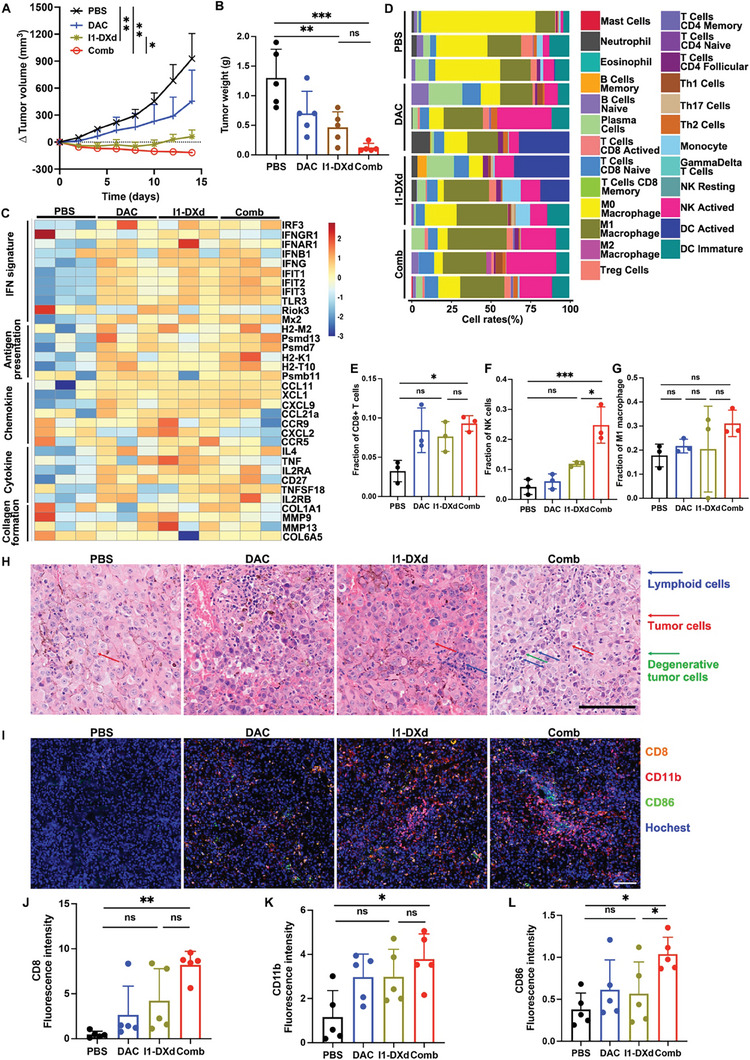
I1‐DXd remodels TME to activate anti‐tumor immunity and synergizes with DAC in immunocompetent mice bearing B16OVA‐hICAM1. (A) Δ tumor volume curve in immunocompetent mice bearing B16OVA‐hICAM1 by tumor volume measurement by caliper. *p < 0.05; **p < 0.01; Bonferroni‐adjusted p value < 0.05 was considered statistically significant. (B) Tumor mass at end point of subcutaneous tumors quantified by weight. **p < 0.01; ***p < 0.001; Bonferroni‐adjusted p value < 0.05 was considered statistically significant. (C) Heat map of IFN signature, antigen presentation, chemokine, cytokine and collagen formation genes in different groups (n = 3 samples per group); (D) Relative proportion bar chart of 25 infiltrated immune cells in each samples according to CIBERSORT algorithm; (E‐G) Quantitative analysis of CD8+T cells (E), NK cells (F) and M1 macrophage cells (G) in different groups. *p < 0.05; ***p < 0.001; Bonferroni‐adjusted p value < 0.05 was considered statistically significant. (H) Representative HE staining images in different groups. Blue arrow indicated lymphoid cells; red arrow indicated tumor cells; green arrow indicated degenerative tumor cells; (I) Representative multiplex immunofluorescence images of tumor samples in different groups for CD8 (pink), CD86 (green) and CD11b (red); (J‐L) Fluorescence intensity analysis of CD8+T cells (J), NK cells (K) and M1 macrophage cells (L) in different groups. *p < 0.05; **p < 0.01; Bonferroni‐adjusted p value < 0.05 was considered statistically significant.

To further investigate the immunoregulatory roles of I1‐DXd and DAC combination on anti‐tumor immunity, we first established an immunocompetent animal model using murine melanoma B16OVA cells transduced with human ICAM1 genes (B16OVA‐hICAM1) (Supplement Figure S3A‐D), which is used to determine the in vivo efficacy of I1‐DXd and DAC combination. The drug and dosage regimes were same with A375‐luc model. As is shown in **Figure** **4**A and Supplement Figure S3E, the B16OVA‐hICAM1 tumor growth was potently suppressed in the Combo group, which is significantly more effective than I1‐DXd or DAC monotherapy. The two‐way ANOVA analysis revealed that the drug effects were statistically different (F = 26.89, p < 0.001) in different groups. Then post hoc with Bonferroni analysis results revealed that Combo group significantly attenuated tumor growth in comparison with PBS and I1‐DXd groups at end point (PBS vs. Combo, Bonferroni‐adjusted p = 0.006; I1‐DXd vs. Combo, Bonferroni‐adjusted p = 0.022). After tumor excision, the results were confirmed by examining terminal tumor weights (Figure 4B). One‐way ANOVA analysis determined significant differences in tumor weight across the study groups (F = 10.95, p < 0.001). Bonferroni‐adjusted post hoc analysis indicated that the I1‐DXd and combination therapy groups had significantly reduced tumor weights compared to the PBS control group (PBS vs. I1‐DXd, Bonferroni‐adjusted p = 0.007; PBS vs. Combo, Bonferroni‐adjusted p < 0.001). Although the tumor weight in the combination therapy group was lower than in the I1‐DXd group, this reduction did not reach statistical significance (0.122g vs. 0.464g; Bonferroni‐adjusted p = 0.758), as shown in Figure 4B.

To further elucidate the immune responses associated with treatment efficacy, whole transcriptional profiles of fresh tumor tissues harvested from different groups (PBS, I1‐DXd, DAC, or Combo group) surveyed by RNA‐Sequencing (RNA‐Seq) were compared. The differentially‐expressed genes (DEGs) among different groups were shown as the volcano map and the top 20 DEGs were presented (Supplement Figure S3F). The KEGG enrichment analysis among different groups were shown in Supplement Figure S4G‐I. In Figure 4C, the transcriptomic results revealed that Combo and I1‐DXd groups substantially upregulated the expression of interferon signature genes (e.g., IFNG, IRF3, IFNGR1), antigen presentation genes (e.g., Mx2, H2‐M2, Psmd13), chemokine and cytokine genes (e.g., CCL1, XCL1, TNF) and downregulated the expression of collagen formation genes (e.g., COL1A1, MMP9). Afterwards, the abundance of different infiltrating immune cells in each sample was evaluated by the CIBERSORT algorithm with the mouse immune cell matrix. As is shown in Figure 4D, the proportions of immune cells exposed several remarkable dissimilarities in I1‐DXd or Combo group when compared with PBS group. Specially, the proportion of CD8+T cells, NK cells and M1 macrophage cells were compared among different groups. One‐way ANOVA analysis demonstrated significant differences in the proportion of CD8+T cells across these groups (F = 6.013, p = 0.019). Further, Bonferroni post hoc analysis revealed that the CD8+T cell proportion in the combination therapy groups was significantly higher than in the PBS control group (Bonferroni‐adjusted p = 0.027; Figure 4E). Similarly, NK cell proportions varied significantly among groups (F = 20.86, p < 0.001), with the combination therapy groups showing a significantly higher proportion of NK cells compared to both the PBS and I1‐DXd groups (PBS vs. combination, Bonferroni‐adjusted p < 0.001; I1‐DXd vs. combination, Bonferroni‐adjusted p = 0.012; see Figure 4F). However, the differences in M1 macrophage cell proportions among the four groups did not reach statistical significance (F = 1.062, p = 0.418). H&E staining tumor tissue sections also revealed that I1‐Dxd or Combo treatment was associated with increased lymphoid cell infiltration and degenerative tumor cells. The increase of infiltrating immune cells was further confirmed by the multiplex immunofluorescence of tumor samples in different treatment groups (Figure 4I and Supplement Figure S4A). The CD8 (pink) was used as a marker of CD8+T cells, CD11b (red) was used as a marker of NK cells and the CD86 (green) was used as a marker of M1 macrophage. In terms of fluorescence intensity, significant differences were observed among groups for CD8+T cells (F = 8.449, p = 0.001), NK cells (F = 4.587, p = 0.016), and M1 macrophage cells (F = 4.424, p = 0.019). Bonferroni post hoc analysis indicated that the fluorescence intensity of CD8+T cells in the combination therapy groups was significantly higher than in the PBS group (Bonferroni‐adjusted p = 0.001; Figure 4J). The same analysis for NK cells and M1 macrophage cells showed that their fluorescence intensities in the combination therapy groups were significantly higher than in the PBS group (NK cells: Bonferroni‐adjusted p = 0.015; Figure 4K. M1 macrophage cells: Bonferroni‐adjusted p = 0.017; Figure 4L). The Combo group demonstrated the highest tumoral proportions of CD8+T cells, NK cells and M1 macrophage cells among all groups. Collectively, these findings indicated that the I1‐DXd and DAC combination exerted a synergistic immunoregulatory effect through accommodating more infiltrated innate and adaptive immune cells to enhance antitumor immunity effect in melanoma TME.

### 2.4 DAC upregulates the expression of ICAM1 via dsDNA accumulation and increase internalization of ICAM1 antibodies

**Figure 5:**
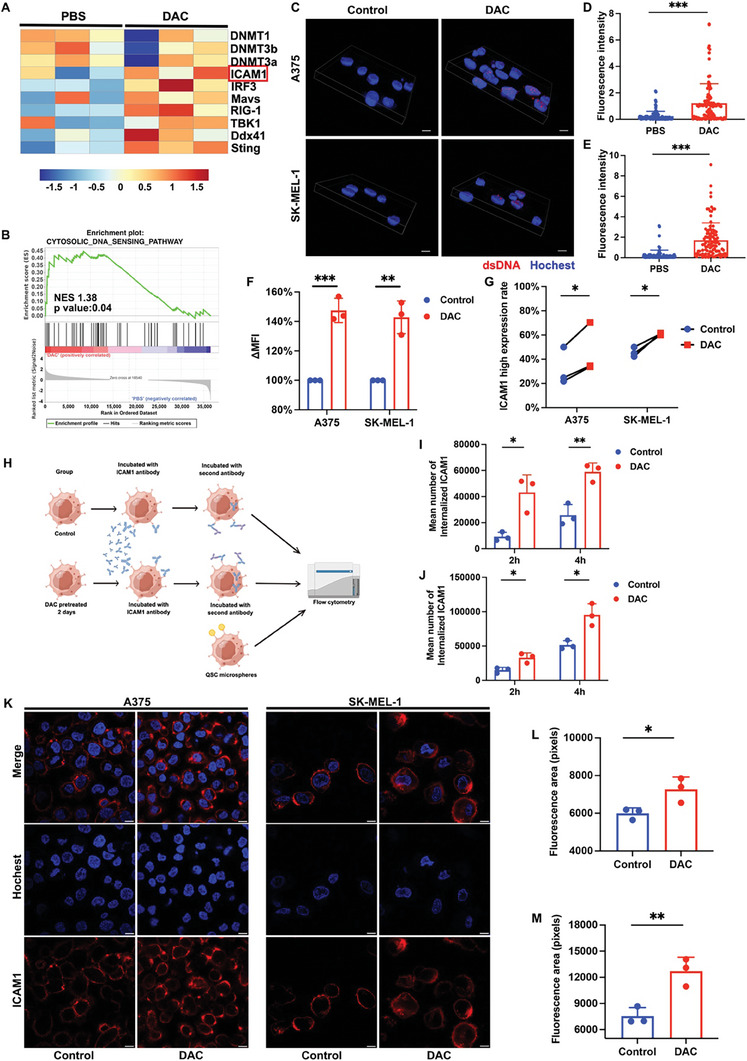
DAC induces ICAM1 expression and increases the internalization of ICAM1 antibody. (A) Heat map showing the down‐regulation of DNMTs and up‐regulation of ICAM1 and dsDNA sensor genes in the DAC group compared with PBS group; (B) GSEA for gene sets associated with cytosolic DNA sensing pathway in DAC group vs. PBS group; (C) Representative images of IF staining of dsDNA in different groups. Scale bar 10 µm; (D‐E) Quantitative fluorescence intensity analysis of dsDNA in A375 cells (D) and SK‐MEL‐1 cells (E). ***p < 0.001; (F) MFI change calculated by flow cytometry after DAC treatment. n = 3 biological replicates; (G) Quantitative analysis of ICAM1 high expression rate after DAC treatment. n = 3 biological replicates. ICAM1 high expression rate was defined by the rate of cells with ICAM1 expression higher than the middle point of the peak of the highest expressing sample in each cell line; (H) Schematic illustration for ICAM1 antibody internalization assay; (I) Quantitative analysis of ICAM1 antibody internalization at 2h and 4 h after DAC treatment in A375 cell (I) and SK‐MEL‐1 cell (J). *p < 0.05; **p <0.01; (K) Representative images of IF staining of ICAM1 antibody at 4 h after DAC treatment. Scale bar 10 µm; (L‐M) Quantitative fluorescence area analysis of tumor cell exposure to ICAM1 antibody at 4 h after DAC treatment in A375 cell (L) and SK‐MEL‐1 cell (M). *p < 0.05; **p <0.01.

Given antigen expression is a key factor for binding and internalizing ADCs.^[^ ^21^ ^]^ As revealed by results of aforementioned transcriptomic data, DAC treatment could effectively up‐regulate ICAM1 expression in response to DNMTs inhibition (**Figure** **5**A). The gene set enrichment analysis revealed that the cytosolic DNA sensing pathway was enriched in DAC group (NES = 1.38; *p* = 0.04), indicating the accumulation of double‐stranded deoxyribonucleic acid (ds‐DNA) (Figure 5B). To further validate dsDNA formation, IF staining with a dsDNA antibody identified that DAC effectively induced the accumulation of dsDNA in both A375 and SK‐MEL‐1 cells (Figure 5C and Supplement Figure S4B). The fluorescence intensity of dsDNA in DAC group was significantly higher than PBS groups in A375 cells (t = 6.455, p < 0.001; Figure 5D) and SK‐MEL‐1 cells (t = 8.263, p < 0.001; Figure 5E). The underlying molecular steps of dsDNA accumulation inducing ICAM1 expression were previously reported through NFkB, JNK and MAPK pathways.^[^ ^22^ ^]^ To systemically evaluate the impact of DAC on the melanoma cell membrane expression of ICAM1 at protein level, A375 and SK‐MEL‐1 cells were pre‐treated with DAC (48h) and ICAM1 membrane expression were evaluated using flow cytometry. The results revealed that the MFI of ICAM1 significantly increased after DAC treatment in A375 cells (t = 9.942, p = 0.001) and SK‐MEL‐1 cells (t = 6.679, p = 0.002; Figure 5F), indicating that DAC treatment significantly increased ICAM1 membrane expression. To make the analysis more comprehensive, the membrane expression of ICAM1 at the single cell level analyzed by Cytobank was further divided into low‐ and high‐expression subgroups. Figure 5G illustrates that the expression rate of ICAM1 significantly increased in A375 cells (t = value: 4.374; p = 0.048) and SK‐MEL‐1 cells (t = 6.254, p = 0.024) following DAC treatment, as determined by paired t‐tests. Moreover, we also investigated the impact of DAC pre‐treatment on the melanoma cell internalization of ICAM1 antibodies. Figure 5H illustrates the procedure for evaluating the internalized antibody quantities. According the flowchart, further analysis using two‐way ANOVA showed statistically significant differences in the effects of DAC treatment between the control and DAC groups for both A375 cells (F = 29.04, p = 0.006) and SK‐MEL‐1 cells (F = 32.66, p = 0.004). Independent t‐tests indicated a significant enhancement in ICAM1 antibody internalization in the DAC‐treated groups compared to controls. In A375 cells, this increase was significant at 2 hours (t = 4.208, p = 0.013; Figure 5I) and became more substantial by 4 hours (t = 5.369, p = 0.005; Figure 5I). Similarly, in SK‐MEL‐1 cells, significant increases were observed at 2 hours (t = 3.877, p = 0.017; Figure 5J) and at 4 hours (t = 4.363, p = 0.012; Figure 5J). At last, IF staining (Figure 5K) visualized DAC disposition substantially increased the exposures of A375 (t = 3.079, p = 0.037; Figure 5L) and SK‐MEL‐1 cells (t = 4.770, p = 0.008; Figure 5M) to ICAM1 antibodies (antibody quantities located in cell membrane and internalized by cell), respectively. These findings strongly support that DAC effectively upregulates ICAM1 expression on melanoma cell surface and enhances internalization of ICAM1 antibodies, providing drug delivery benefits for ICAM1‐ADCs.

### 2.5 DAC potentiates melanoma cytotoxicity of I1‐DXd

**Figure 6:**
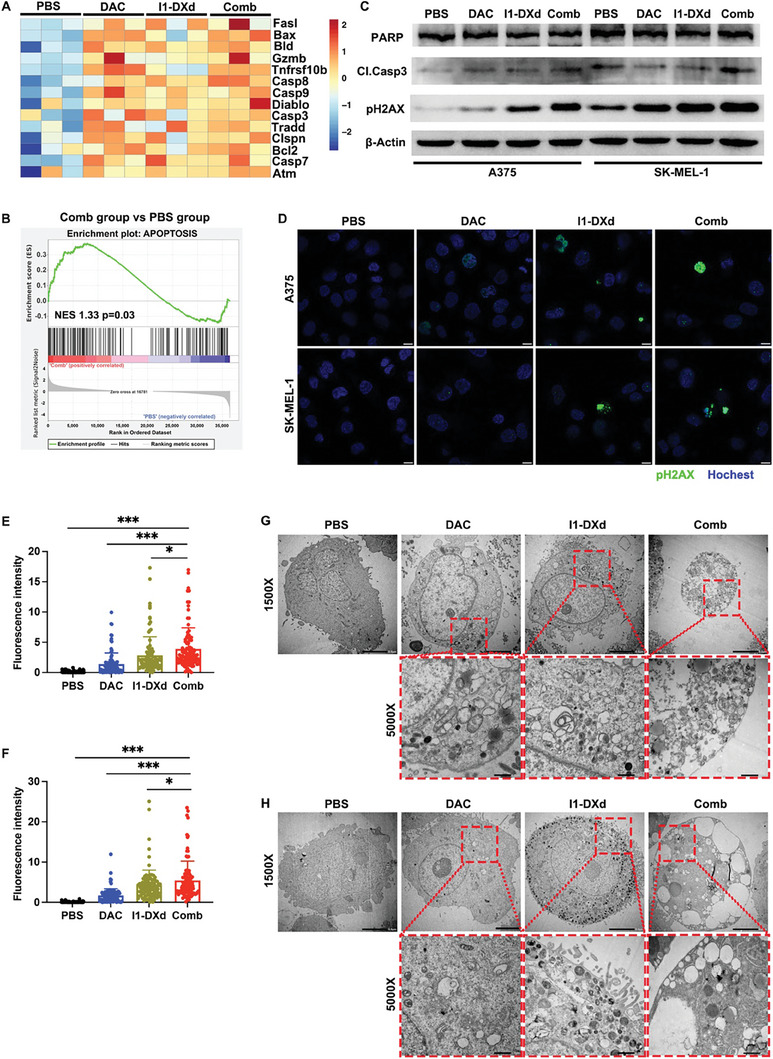
DAC enhances the anti‐tumor efficacy of I1‐DXd by synergistically inducing apoptotic cancer cell death. (A) Heat map of apoptosis‐related genes expression in different groups; (B) GSEA for gene sets associated with apoptosis pathway in the Combination group vs. PBS group; (C) Western blots showing the DNA damage and apoptosis response followed by I1‐DXd monotherapy or combination treatment with DAC. β‐Actin is the loading control; (D) Representative images of IF staining of pH2AX in different groups. Scale bar 10 µm; (E‐F) Fluorescence intensity analysis of pH2AX in different groups in A375 cell (E) and SK‐MEL‐1 cell (F) (n = 100 cells per group); *p < 0.05; ***p < 0.001; Bonferroni‐adjusted p value < 0.05 was considered statistically significant. (G‐H) Representative TEM image of A375 (G) and SK‐MEL‐1 (H) after different treatment for 24h.

Given that the mechanism of action (MoA) of I1‐DXd primarily acts on inducing apoptotic cancer cell death via its cytotoxic payload of DXd, a DNA topoisomerase inhibitor, we next investigated the impact of DAC on the sensitivity of melanoma cell to I1‐DXd treatment. In consistence with the outstanding inhibitory effects on melanoma tumor growth, we observed that Combo and I1‐DXd groups potently activated apoptosis‐related genes in our transcriptomic analysis (**Figure** **6**A). The apoptosis pathway also was enriched in both I1‐DXd and Combo group by KEGG pathway enrichment analysis (I1‐DXd vs. PBS, NES 1.46, p = 0.01; Combo vs. PBS, NES 1.33, p = 0.03; Figure 6B). To validate our transcriptomic results at protein level, we performed western‐blot and identified substantially increased levels of cleaved caspase‐3 and pH2aX, two established markers of cell apoptosis, in the Combo group. The upregulated pH2aX expression in the Combo group was also visually confirmed by IF staining in both A375 and SK‐MEL‐1 cells (Figure 6D). The one‐way ANOVA analysis indicated significant differences in the fluorescence intensity of pH2aX across various groups in A375 cells (F = 44.76, p < 0.001). Subsequent Bonferroni post hoc analysis revealed that the fluorescence intensity of pH2aX in the combination therapy groups was significantly higher than in the PBS, DAC, and I1‐DXd groups (PBS vs. Combo, Bonferroni‐adjusted p < 0.001; DAC vs. Combo, Bonferroni‐adjusted p < 0.001; I1‐DXd vs. Combo, Bonferroni‐adjusted p = 0.015; Figure 6E). Similarly, in SK‐MEL‐1 cells, the fluorescence intensity of pH2aX varied significantly among groups (F = 59.24, p < 0.001), with the combination therapy groups showing significantly greater intensity compared to the PBS, DAC, and I1‐DXd groups (PBS vs. Combo, Bonferroni‐adjusted p < 0.001; DAC vs. Combo, Bonferroni‐adjusted p < 0.001; I1‐DXd vs. Combo, Bonferroni‐adjusted p = 0.044; Figure 6F). The apoptotic morphologies of melanoma cells induced by I1‐DXd or in combination with DAC were observed intuitively by transmission electron microscope (TEM) (Figure 6G‐H). These above findings support that DAC potently sensitizes melanoma cells to I1‐DXd treatment by improving apoptotic cancer cell death.

### 2.6 DAC enhances tumor penetration of I1‐DXd

**Figure 7:**
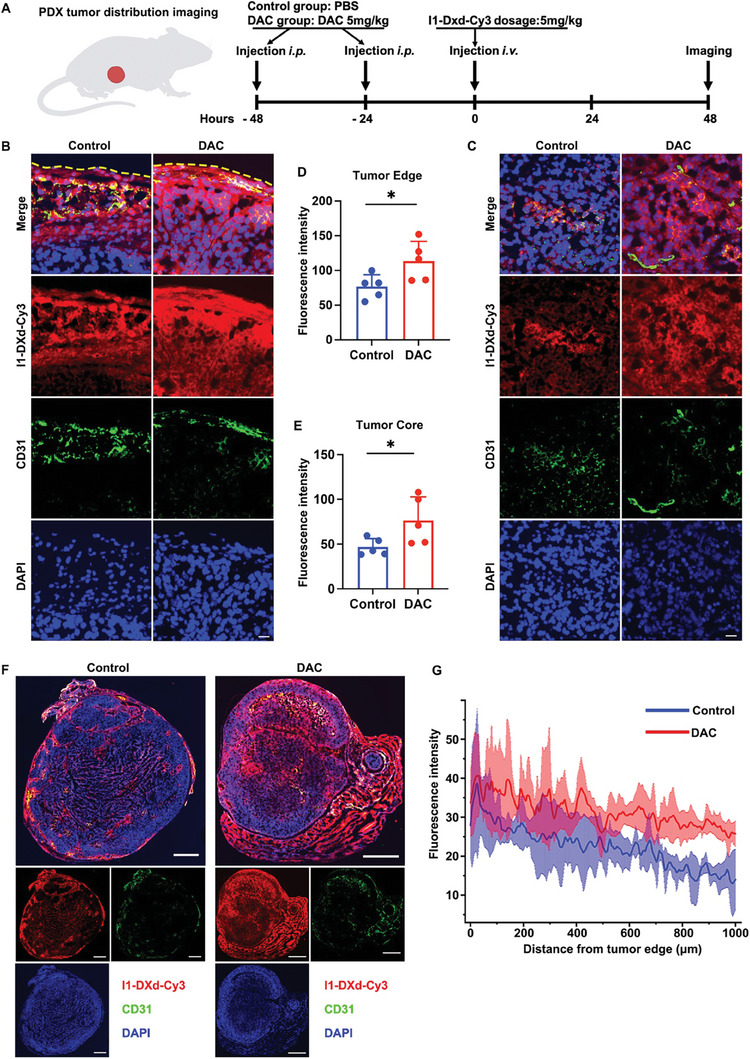
DAC enhances tumor penetration of I1‐DXd. (A) Schematic design of I1‐DXd biodistribution in PDX tumor; (B) Representative fluorescence images of tumor edge; yellow dotted line indicated the margin of tumor; (C) Representative fluorescence images of tumor core; (D) Quantitative analysis of fluorescence intensity in tumor edge in control or DAC group (n = 5 per group). *p < 0.05; (E) Quantitative analysis of fluorescence intensity in tumor core in control or DAC group (n = 5 per group). *p < 0.05; (F) Representative fluorescence images of the whole tumor in different groups; (G) Euclidean distance map of I1‐DXd‐Cy3 distribution in different groups (n = 5 per group).

Inspired by the RNA‐seq results (Figure 4C and Figure 5A) that DAC could upregulate ICAM1 expression and reprogram the tumor microenvironment (increasing infiltrating immune cells, upregulating chemokines and downregulating collagen formation genes), we speculated that DAC may improve tumor penetration of I1‐DXd. Thus, we studied the impact of DAC treatment on the tumoral biodistribution of the fluorophore Cy3‐labeled I1‐DXd (I1‐DXd‐Cy3) in PDX model (**Figure** **7**A). The whole tumor imaging captured the overall I1‐DXd‐Cy3 distribution after 48h following intravenous delivery at the same doses as the efficacy study. Nuclear staining was performed using Hoechst 33258 and vasculature staining was performed by CD31. We calculated the fluorescence intensity from tumor edge to tumor core. The independent t‐test demonstrated that DAC treatment significantly increased the fluorescence intensities of I1‐DXd‐Cy3, both at the tumor edge (t = 2.484, p = 0.037; Figures 7B‐C) and in the tumor core (t = 2.358, p = 0.046; Figures 7D‐E), indicating that DAC effectively enhances solid tumor penetration of I1‐DXd‐Cy3. The distance map further confirms that I1‐DXd‐Cy3 achieved a substantially better tumoral penetration with assistance from DAC treatment (Figure 7F‐G).

**Figure 8:**
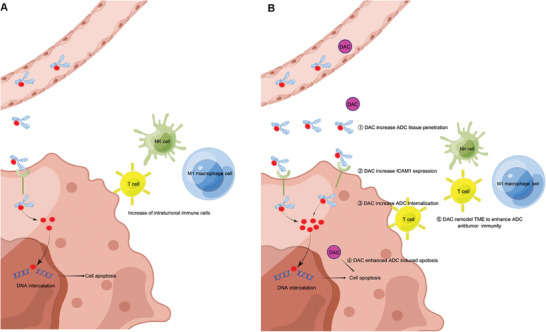
Schematic depicting the antitumor mechanisms of I1‐ADC monotherapy (A) and the synergistic antitumor mechanisms of I1‐ADC in combination with DAC (B).

Lastly, we concluded this study by drawing a schematic diagram of the anti‐tumor mechanism of I1‐DXd monotherapy and synergistic antitumor mechanisms of I1‐ADC in combination with DAC (**Figure** **8**).

## 3 Discussion

Malignant melanoma is a highly aggressive tumor with limited treatment options, especially after failure of immune therapy.^[^ ^23^ ^]^ Although ADC has emerged as paradigm‐shifting therapeutic strategy for many cancers including breast and gastric cancers,^[^ ^24^ ^]^ there is no ADC clinically approved for melanoma currently. To address this unmet medical need and to respond to the concerns noted in the real‐world practice, we report the development of rationally designed ADCs targeting ICAM1 as a potent targeted therapeutic for treating melanoma through direct killing of malignant cells in multiple cell line‐derived xenografts (CDX) and PDX models of melanoma. Further, we discovered that ICAM1‐ADC could reprogram TME into an active immune microenvironment in the immunocompetent xenograft model. The dual therapeutic benefits of ICAM1‐ADC might pave the road for clinical development of ADC for melanoma. Furthermore, we propose a rational therapeutic strategy that combines ADC and epigenetic drug DAC and elucidate the possible synergistic mechanism of this drug combination.

Combination therapy is a widely acknowledged strategy to increase the likelihood of achieving complete remission and cure by cooperating through diverse mechanisms of action, particularly when dealing with the complexities of tumor heterogeneity.^[^ ^11^ ^]^ To date, therapeutic benefits and underlying biomechanism(s) of ADCs in combination with other targeted therapeutics remain elusive. In this study, we identified the partner of choice with synergy in antitumor activity from the perspective of epigenetics by performing an unbiased screening of 29 epigenetic chemical compounds. After screening, we identified DNA methylation inhibitor (DAC) as an effective choice of combination with “magic bullets” ADC for melanoma. DNA methylation, as the main epigenetic modification and a well‐known epigenetic marker, is modified by DNA methyltransferase (DNMTs). Previous study has reported that aberrant DNA methylation was significantly associated with poorer prognosis ^[^ ^14^, ^25^ ^]^ and more resistant to chemotherapy and BRAF inhibitors in melanoma.^[^ ^26^ ^]^ The reversible nature of DNA methylation makes it a desirable therapeutic target of combination treatment. Previous clinical trials for melanoma have shown the safety of DAC in combined with other drugs such as temozolomide ^[^ ^16^ ^]^ and interleukin‐2^[^ ^27^ ^]^). In this study, we specifically investigated the synergy between I1‐DXd and DAC, demonstrating superior in vivo efficacy than I1‐DXd monotherapy in multiple preclinical models of melanoma. Additionally, DAC did not add to the toxicity of I1‐DXd therapy when combined.

Rational drug combinations require the understanding of how DAC works in synergy with I1‐DXd. Mechanistically, we discovered that combination with DAC generates five major advantages for improving the in vivo efficacy of I1‐DXd as follows: (1) I1‐DXd monotherapy reprogrammed TME to boost anti‐tumor immunity in synergy with DAC; (2) DAC upregulates ICAM1 expression on melanoma cell surface via dsDNA accumulation; (3) DAC increases the cellular internalization of ICAM1‐ADC; (4) DAC sensitizes melanoma tumors to I1‐DXd treatment via promoting apoptosis; (5) DAC enhances tumor penetration of I1‐DXd in vivo. The anti‐tumor efficacy of ADC largely depends on antigen expression, internalization of ADC within tumor cells, and the potency of the cytotoxic payload.^[^ ^28^ ^]^ In this study of in vitro experiments, we found that DAC could significantly promote the accumulation of dsDNA. Previous study reported that the accumulation of dsDNA could activate the cytosolic DNA sensing by the cGAS‐STING pathway and thus upregulate the expression of IRF3,^[^ ^29^ ^]^ which could directly bound to the ICAM1 promoter to induce ICAM1 expression.^[^ ^30^ ^]^ High ICAM1 expression in melanoma correlates with poor outcomes.^[^ ^14^ ^]^ However, several ADC clinical research revealed that antigen loss is a predominant cause for acquired resistance of ADC treatment.^[^ ^31^ ^]^ The DAISY trial underscored this by showing a 65% reduction in HER2 expression in patients experiencing tumor progression with T‐DXd, a HER2‐targeted ADC.^[^ ^31^ ^]^ Similarly, the absence of TROP2 was linked to initial resistance to sacituzumab govitecan, a TROP2‐targeted ADC.^[^ ^32^ ^]^ Addressing antigen loss, combination therapy with ADCs presents a promising strategy.^[^ ^11^ ^]^ For instance, Osimertinib, an EGFR tyrosine kinase inhibitor, has been found to synergize with HER3‐targeted ADCs by boosting HER3 expression and enhancing ADC uptake in lung cancer cells.^[^ ^33^ ^]^ Concurrently, DAC functioning through a distinct mechanism, may complement I1‐DXd, potentially mitigating intrinsic ADC resistance. This integrated approach suggests a viable pathway to counteract antigen loss and improve response rates in ADC treatments. Besides, DAC also significantly increases the number of internalized ICAM1 antibodies. To sum up, the increase of membrane expression and internalization of ICAM1 could significantly increase the antibody exposure of tumor. On the other hand, DAC also significantly promotes the apoptotic cancer cell death by I1‐DXd in comparison with I1‐DXd monotherapy, as evidenced by RNA sequencing, western‐blot, TEM and morphology of H&E staining slices. DXd, as the cytotoxic payload of I1‐DXd, is a DNA topoisomerase I (TOP1) inhibitor with higher anti‐tumor activity and cell membrane permeability than SN‐38. The inhibition of TOP1 has been reported to induce apoptosis of tumors.^[^ ^34^ ^]^ Our findings also provide the first experimental evidence that DXd‐based ADCs exhibit potent anti‐tumor efficacy against melanoma.

Another important finding of this work is the TME remodeling effect of I1‐DXd monotherapy or combined with DAC. TME plays a key role in malignancy development, progression, and metastasis orchestrated by the intricate crosstalk between cellular and non‐cellular components.^[^ ^35^ ^]^ The mechanism of melanoma immune evasion after immune therapy included suppressing ability of antigen presentation, decreasing mutational burden and upregulating of coinhibitory receptors.^[^ ^36^ ^]^ Previous study revealed that DAC alone could upregulate immunostimulatory genes (Cxcl1, Cxcl10, IRF7 etc.) and enhance lymphocyte migration and function.^[^ ^37^ ^]^ In this study, we investigated the biological functions of I1‐DXd single agent or combined with DAC on anti‐tumor immune responses using immunocompetent mice bearing B16OVA‐hICAM1 tumor. As demonstrated by CIBERSORT analysis and multiplex immunofluorescence of tumor tissue, the proportions of infiltrating anti‐tumor immune cells (CD8+T cells, NK cells and M1 macrophage cells) in TME are significantly increased after the treatment of I1‐DXd. More importantly, the I1‐DXd mediated TME amelioration was significantly enhanced when combined with DAC. The characterization variability of TME also provided insight into the immunomodulatory properties after I1‐DXd and DAC combination treatment relying on the activation of a diverse array of antitumor immune cascades. It is conceivable that the traits together contribute to the ADC/DAC combination‐mediated enhanced infiltrating anti‐tumor immune cells might revert resistance to the benefit of immune checkpoint blockade.

The tumor penetration depth of ADC was multi‐factorially determined by target density, internalization rate, antibody molecular weight, drug dose, and tumor physiology. Based on the ameliorated TME remodeling and increasing tumoral exposure of ICAM1 antibodies, the impact of DAC on tumor penetration of I1‐DXd was further evaluated in the PDX model of melanoma. We found that DAC pretreatment helped I1‐DXd to readily extravasate and significantly enhanced tumor penetration of I1‐DXd, especially in the tumor core. Such improved tumor penetration ability of ICAM1‐ADC induced by DAC may yield greater efficacy and improve clinical success rates for ADCs, since solid tumor tissue penetration holds the key for their clinical efficacy.^[^ ^38^ ^]^ Our findings provide in vivo evidence that DAC pretreatment improves tumor penetration of ADCs.

To further enhance this study, more efforts could be taken into action in four following aspects: (1) although our data show that epigenetic inhibitor DAC significantly enhanced in vivo efficacy of ICAM1‐ADCs by providing diverse advantages from drug delivery to TME remodeling, their key molecular steps remain needs to be identified in more detailed biomechanism investigations. (2) PDX models, being directly transplanted into mice, offer a greater degree of heterogeneity and variability compared to CDX. To enhance the robustness of our findings, we have utilized two complementary CDX models (A375‐luc in immunodeficient and B16OVA in immunocompetent mice) to address the variability encountered with the PDX model. (3) given that mice exhibit a higher tolerance to various ADC payloads compared to humans,^[^ ^39^ ^]^ the safety and toxicity profile of coadministration of epigenetic drugs in combination with ADC have yet to be clearly understood and further studies are needed in large animals and non‐human primates, which more accurately mirror human clinical responses and tolerances. (4) we are working on next‐generation ADCs featuring dual payloads (cytotoxic payload and epigenetic inhibitor) that simultaneously deliver two cooperative agents into same target tumor cells in our future study. The preliminary results showed encouraging prospects.

## 4 Conclusion

In summary, our study provides critical insights into the development of promising ADC candidates for melanoma‐targeted therapy. Furthermore, this study revealed that DAC could enhance antitumor efficacy of ICAM1‐ADC with different mechanisms. These findings provide supporting rationale for ICAM1‐ADC as candidate for melanoma and pursue DAC as a combination partner. This combination could also open a new avenue for ADC‐based therapeutics for treating other intractable malignancies.

## 5 Experimental Section

### Cell lines and cell culture

Human melanoma cell lines A375, C32 and SK‐MEL‐1 were obtained from the American Type Culture Collection (ATCC). The normal human embryonic kidney HEK 293T cell was maintained in our lab. Mouse melanoma cell line B16OVA was kindly provided by Prof. Hao Chang at HIM. To construct the hICAM1+ mouse melanoma cell line, the full‐length human ICAM1 gene (NM_000201) was retro‐virally introduced into B16‐OVA cells (B16OVA‐hICAM1) with lentivirus vector GV492. Then, cell pools stably expressing hICAM1 were selected and individual clones were isolated by single‐cell sorting with flow cytometry. An empty vector (pQCXIN, Clontech) was also retro‐virally introduced as negative control (B16OVA‐mock).

All cell lines were cultured with the appropriate media (RPMI 1640 for A375, MEM supplemented with MEM non‐essential amino acids solution for SK‐MEL‐1, DMEM for C32, 293T and B16OVA) supplemented with 10% heat‐inactivated FBS at 37 °C in a humidified incubator with 5% (vol/vol) CO_2_.

### Evaluating cell surface ICAM1 expression and its internalization

The ICAM1 expression levels were quantified by Beckman Coulter's CytoFLEX LX Flow Cytometer with ICAM1 antibodies (Cat 353106, BioLegend, San Diego, CA) as previously detailed.^[^ ^40^ ^]^ The internalization assay was performed in melanoma and 293T cells following our reported protocol.^[^ ^10^ ^]^ Briefly, the detached cells were first incubated with unconjugated anti‐human ICAM1 antibodies (Cat 322702, BioLegend, San Diego, CA) for 30min at 4 °C. After washing, the primary ICAM1 antibodies bound on cell membranes were allowed to be internalized for different time points (0min, 30min, 60min, 120min, 240min at 37 °C). A secondary PE‐conjugated anti‐mouse IgG (BioLegend, San Diego, CA) was added to the stained cell for 30min and then rinsed by PBS, fixed by paraformaldehyde (4%) for flow cytometry. The internalization efficiency was calculated by the formula (1‐mean cell fluorescence intensity (t = incubation time)/mean cell fluorescent intensity (t = 0 min) × 100%. The extent of internalization was presented with internalization curve which was generated by internalization efficiency at different time points. As previously reported by us, the Quantum™ Simply Cellular® (QSC) microspheres kit (Bangs Laboratories, IN, USA) was applied to quantify the cell surface expression of ICAM1 on various cancer cell lines.^[^ ^41^ ^]^

### Fluorescence imaging by confocal microscopy

PE conjugated ICAM1 antibody was incubated with cells at 37 °C for 45min, then washed, fixed and then analyzed by confocal microscopy (Nikon A1 HD25; Nikon, Japan). For internalization experiments, detached cells were incubated with PE‐conjugated ICAM1 antibody for different time points, then processed and analyzed as described in “Internalization assay”. To analyze the accumulation of dsDNA and pH2AX, the dsDNA antibody (Cat ab27156, Abcam, Cambridge, UK) or pH2AX antibody (Cat 2577, Cell signaling, Bionordika, Stockholm, Sweden) was incubated with cells, then washed. The secondary PE‐conjugated anti‐mouse IgG (BioLegend, San Diego, CA; diluted 1: 1000) or secondary FITC‐conjugated anti‐mouse IgG (Cat A22110; Abbkine, China) was incubated for 1 hour at room temperature. Next, the cells were washed thrice with PBS to remove unbound secondary antibody. Cell nuclei were counterstained with Hoechst 33258 in PBS for 10 minutes at room temperature.

### Western blot

Cell lines were lysed with RIPA lysis buffer. SDS‐page and western blot were performed using standard protocols.

### Preparation of antibody drug conjugates and evaluating cell cytotoxicity of ADCs

The payloads of the two ICAM1‐ADCs were deruxtecan (DXd) and monomethyl auristatin E(MMAE). I1‐DXd and I1‐MMAE were prepared (GLP grade) by MabPlex (Yantai, China) via covalently conjugated ICAM1 antibody (R6.5c, GeneScript) with corresponding ADC linker and payload combinations (MC‐GGFG‐DXd and MC‐VC‐PAB‐MMAE). The average drug‐to‐antibody ratios (DARs) of I1‐DXd and I1‐MMAE were measured by using hydrophobic interaction chromatography (HIC). In the cytotoxic assay, the melanoma and 293T cells were seeded in a 96‐well plate at a density of 2000–5000 cells. After overnight incubation, the cell culture medium was replaced with the medium containing either ICAM1‐ADCs or control chemo drugs at serial diluted concentrations. After 96h, cell cytotoxicity was determined by using a CCK‐8 kit (KeyGEN Biotech, China) following the manufacturer's protocol. The absorbance at 450nm was measured with an ELISA browser (Bio‐Tek EL 800, USA). IC50s were calculated by GraphPad Prism v9 (GraphPad Software, Inc., La Jolla, CA).

### In vivo efficacy of ICAM1 ADCs

All animal experiments were performed according to the protocols approved by the Institutional Animal Care and Use Committee (IACUC) of Zhejiang Cancer Hospital.

To generate CDX, A375‐luc cells (2 × 10^6^) were injected subcutaneously with matrigel (Corning, 100 µL) in the flanks of 6‐week‐old male nude mice. To generate melanoma PDX, fresh melanoma fragments were transplanted subcutaneously into the right flank of anaesthetized nud mice. To generate immunocompetent subcutaneous xenograft model, C57BL/6 mice (aged 4–6 weeks) were inoculated with 2 × 10^6^ B16OVA‐hICAM1 cells into the right flank by subcutaneous (s.c.) injection. Then the tumor‐bearing mice were randomly divided into different treatment groups (n ≥ 5 per group). In the in vivo study, the paclitaxel, ICAM1 antibody, I1‐DXd or I1‐MMAE were injected at an equivalent dosage of 5 mg/kg per week via tail vein injection with a total of 2 courses. The DAC was injected at a dosage of 5 mg/kg 3 times weekly via intraperitoneal injections with a total of 6 courses. The DAC dosage used in mice was converted from dose treating human hematopoietic malignancies (15 mg/m2/d). The dose conversion was based on the FDA recommendation (https://www.fda.gov/downloads/drugs/guidances/ucm078932.pdf) and previous literature.^[^ ^42^ ^]^ Tumor growth was monitored twice weekly by two‐dimensional measurements using a vernier caliper. The tumor size was monitored. Animals were also monitored closely for signs of discomfort or pain and weight. Tumor volume was estimated according to the formula: tumor volume (mm^3^) = tumor width^2^ × tumor length × 0.5. Animals were euthanized at the end of the study, or when tumors reached 1500 mm^3^. The tumor and major organs from different groups were embedded, cutted into 4‐µm‐thick sections and hematoxylin and eosin (H&E) stained with hematoxylin and eosin (H&E) and immunohistochemistry (IHC). Subsequently, bright‐field images were taken using SlideView VS200 (Olympus, Japan) for histological examination. The serum levels of alanine aminotransferase (ALT), aspartate aminotransferase (AST), blood concentration of creatinine (BUN) and creatinine (Cre) were determined with biochemical analyzer Hitachi‐7180 (Hitachi, Yokohama, Japan).

### RNA sequencing and analysis

Total RNA from excised tumors in tumor‐bearing immunocompetent C57BL/6 mice was isolated using TRIzol (Thermofisher, 15596018) following the manufacturer's procedure. The total RNA quantity and purity were assessed by Bioanalyzer 2100 and RNA 6000 Nano LabChip Kit (Agilent, CA, USA, 5067‐1511) with RIN number >7.0. The RNA samples were then used to construct sequencing library. After total RNA was extracted, mRNA was purified with two rounds of purification and then mRNA was fragmented into short fragments using divalent cations under elevated temperature (Magnesium RNA Fragmentation Module (NEB, cat.e6150, USA) under 94 °C; 5–7min). The cleaved RNA fragments were reverse‐transcribed to produce cDNA. The average size of cDNA fragments in the library was 300 ± 50 bp. At last, the 2 × 150 bp paired‐end sequencing (PE150) was performed on an Illumina Novaseq™ 6000 (LC‐Bio Technology CO., Ltd., Hangzhou, China) following the vendor's recommended procedure. Reads obtained from the sequencing machines includes raw reads containing adapters or low‐quality bases which will affect the following assembly and analysis. Thus, to get high‐quality clean reads, reads were further filtered by Cutadapt (https://cutadapt.readthedocs.io/en/stable/, version: cutadapt 1.9).

Differentially expressed genes (DEGs) analysis was performed by R package edgeR. The genes with the parameter of false discovery rate (FDR) < 0.05 and absolute fold change > 2 were considered differentially expressed genes. Differentially expressed genes were then subjected to enrichment analysis of KEGG pathways. Advanced Heatmap Plots to visualize specific gene expressions were performed by OmicStudio tools (https://www.omicstudio.cn). Based on the RNA sequence data, the infiltrating proportion of immune cells in each sample was deconvoluted by “CIBERSORT” algorithm ^[^ ^43^ ^]^ with the mouse immune cell matrix.^[^ ^44^ ^]^

### Fluorescence histology for imaging ADC tumor distribution

The tumor distribution of fluorescent ADC was assessed by fluorescence imaging. Briefly, the PDX mice were pretreated with DAC (5mg/kg, i.p.; day 1 and day 3) or PBS when the tumor volume was about 250mm^3^. Then ICAM1‐DXd‐Cy3 was administered via tail‐vein injection in PDX mice at a dosage of 5mg/kg mouse weight. At 48h post injection, mice were sacrificed and tumors were then resected. The resected tumors were optimal cutting temperature (OCT)‐embedded and flash frozen in liquid nitrogen, then were sectioned into 10µm‐thick slices using a cryostat. Before imaging, tumor slices were stained with anti‐mouse CD31 (BioLegend, 102402) overnight at 4 °C and incubated with fluorophore‐conjugated secondary antibodies (Alexa Fluor 488 goat anti‐rabbit IgG; 1: 500; Abbkine, Wuhan, China) for 30 min. Nucleus was stained by Hoechst 33258 (Hoechst) for 10 min. Microscopy was performed using fluorescence microscope (Nikon A1 HD25; Nikon, Japan) with a 20× objective. The images were obtained by stitching smaller images with the Olympus software and were analyzed using ImageJ image analysis software. The central part of the whole tumor region was defined as the tumor core and the tumor edge was defined as within 200µm interface between tumor and non‐malignant tissue.

### Statistical analysis

Quantitative data are presented as the means ± standard deviations (SD). Statistical evaluations were conducted using the Pearson correlation test to assess correlations. For comparisons between two independent or two paired samples, the independent samples t‐test and paired t‐test were utilized, respectively. For analyses involving multiple comparisons, a two‐way mixed ANOVA was employed, and in instances of significant differences, one‐way ANOVA was applied to further investigate these disparities. Post hoc with Bonferroni analyses were conducted where deemed necessary to adjust for multiple comparisons. When multiple comparisons were applied, the adjusted p value was measured by Bonferroni correction.^[^ ^45^ ^]^ The Bonferroni‐adjusted p value was defined as the uncorrected p value multiplied by the number of comparisons and Bonferroni‐adjusted p value < 0.05 was considered statistically significant.^[^ ^46^ ^]^ This study designated the change in tumor volume from baseline (Δ tumor volume) as the primary endpoint and selected the final tumor weight as the secondary endpoint to assess antitumor activity. Statistical analysis was performed using Stata 13 or GraphPad Prism v9 (GraphPad Software, Inc., La Jolla, CA). The figures were performed using GraphPad Prism v9 and Origin 8.0 (Origin Software, Inc., OriginLab, USA) software. The p value <0.05 was considered statistically significant.

## Conflict of Interest

P.Z. and P.G. are co‐inventors of a patent application filed by Zhejiang Provincial People's Hospital and Hangzhou Institute of Medicine, Chinese Academy of Sciences. X.L. and Y.D. are shareholders of MabPlex. The other authors have declared no conflicts of interest.

## Author Contributions

P Z, L Y, D H and P G. These authors contributed equally to this work and are co‐corresponding authors. P.Z., CJ. Tao., T. S., JM. F., DW. H., L.Y. and P.G. designed and wrote the paper. P.Z., C.T., and Y.L. contributed equally to this work. YJ. D. and XF. L. constructed the antibody‐drug conjugates. P.Z., CJ. Tao. and PJ. L. performed the experiments. Y. L. analyzed the experimental data. Y.X. and X.W. analyzed and supervised the immunohistochemical experiments. All authors read and approved the manuscript.

## Supporting information

Supporting Information

## Data Availability

The data that support the findings of this study are available from the corresponding author upon reasonable request.

## References

[1] G. V. Long , S. M. Swetter , A. M. Menzies , J. E. Gershenwald , R. A. Scolyer , Lancet 2023, 402, 485. 37499671 10.1016/S0140-6736(23)00821-8

[2] A. Ackerman , O. Klein , D. F. McDermott , W. Wang , N. Ibrahim , D. P. Lawrence , A. Gunturi , K. T. Flaherty , F. S. Hodi , R. Kefford , A. M. Menzies , M. B. Atkins , G. V. Long , R. J. Sullivan , Cancer 2014, 120, 1695. 24577748 10.1002/cncr.28620

[3] S. Modi , C. Saura , T. Yamashita , Y. H. Park , S. B. Kim , K. Tamura , F. Andre , H. Iwata , Y. Ito , J. Tsurutani , J. Sohn , N. Denduluri , C. Perrin , K. Aogi , E. Tokunaga , S. A. Im , K. S. Lee , S. A. Hurvitz , J. Cortes , C. Lee , S. Chen , L. Zhang , J. Shahidi , A. Yver , I. Krop , D. E.‐B. Investigators , N. Engl. J. Med. 2020, 382, 610.31825192

[4] S. Sandhu , C. M. McNeil , P. LoRusso , M. R. Patel , O. Kabbarah , C. Li , S. Sanabria , W. M. Flanagan , R. F. Yeh , F. Brunstein , D. Nazzal , R. Hicks , V. Lemahieu , R. Meng , O. Hamid , J. R. Infante , Invest New Drugs 2020, 38, 844. 31385109 10.1007/s10637-019-00832-1

[5] P. A. Ott , A. C. Pavlick , D. B. Johnson , L. L. Hart , J. R. Infante , J. J. Luke , J. Lutzky , N. E. Rothschild , L. E. Spitler , C. L. Cowey , A. R. Alizadeh , A. K. Salama , Y. He , T. R. Hawthorne , R. G. Bagley , J. Zhang , C. D. Turner , O. Hamid , Cancer 2019, 125, 1113. 30690710 10.1002/cncr.31892

[6] P. Guo , J. Yang , D. R. Bielenberg , D. Dillon , D. Zurakowski , M. A. Moses , D. T. Auguste , J Control Release 2017, 263, 57. 28341549 10.1016/j.jconrel.2017.03.030 PMC5603366

[7] T. M. Bui , H. L. Wiesolek , R. Sumagin , J Leukoc Biol 2020, 108, 787. 32182390 10.1002/JLB.2MR0220-549R PMC7977775

[8] I. M. Min , E. Shevlin , Y. Vedvyas , M. Zaman , B. Wyrwas , T. Scognamiglio , M. D. Moore , W. Wang , S. Park , S. Park , S. Panjwani , K. D. Gray , A. B. Tassler , R. Zarnegar , T. J. Fahey 3rd , M. M. Jin , Clin. Cancer Res. 2017, 23, 7569. 29025766 10.1158/1078-0432.CCR-17-2008 PMC5732861

[9] P. Guo , J. Huang , B. Zhu , A. C. Huang , L. Jiang , J. Fang , M. A. Moses , Sci. Adv. 2023, 9, eabq7866. 37146146 10.1126/sciadv.abq7866 PMC10162665

[10] P. Zhang , C. Tao , T. Shimura , A. C. Huang , N. Kong , Y. Dai , S. Yao , Y. Xi , X. Wang , J. Fang , M. A. Moses , P. Guo , iScience 2023, 26, 107272. 37520726 10.1016/j.isci.2023.107272 PMC10371847

[11] J. Z. Drago , S. Modi , S. Chandarlapaty , Nat. Rev. Clin. Oncol. 2021, 18, 327. 33558752 10.1038/s41571-021-00470-8 PMC8287784

[12] D. Hanahan , Cancer Discov 2022, 12, 31. 35022204 10.1158/2159-8290.CD-21-1059

[13] S. M. Castellino , Q. Pei , S. K. Parsons , D. Hodgson , K. McCarten , T. Horton , S. Cho , Y. Wu , A. Punnett , H. Dave , T. O. Henderson , B. S. Hoppe , A. M. Charpentier , F. G. Keller , K. M. Kelly , N. Engl. J. Med. 2022, 387, 1649. 36322844 10.1056/NEJMoa2206660 PMC9945772

[14] M. Gassenmaier , M. Rentschler , B. Fehrenbacher , T. K. Eigentler , K. Ikenberg , C. Kosnopfel , T. Sinnberg , H. Niessner , H. Bosmuller , N. B. Wagner , M. Schaller , C. Garbe , M. Rocken , Am J Pathol 2020, 190, 2155. 32679231 10.1016/j.ajpath.2020.07.002

[15] S. J. Hogg , P. A. Beavis , M. A. Dawson , R. W. Johnstone , Nat Rev Drug Discov 2020, 19, 776. 32929243 10.1038/s41573-020-0077-5

[16] H. A. Tawbi , J. H. Beumer , A. A. Tarhini , S. Moschos , S. C. Buch , M. J. Egorin , Y. Lin , S. Christner , J. M. Kirkwood , Ann. Oncol. 2013, 24, 1112. 23172636 10.1093/annonc/mds591 PMC3603441

[17] A. Tsherniak , F. Vazquez , P. G. Montgomery , B. A. Weir , G. Kryukov , G. S. Cowley , S. Gill , W. F. Harrington , S. Pantel , J. M. Krill‐Burger , R. M. Meyers , L. Ali , A. Goodale , Y. Lee , G. Jiang , J. Hsiao , W. F. J. Gerath , S. Howell , E. Merkel , M. Ghandi , L. A. Garraway , D. E. Root , T. R. Golub , J. S. Boehm , W. C. Hahn , Cell 2017, 170, 564. 28753430 10.1016/j.cell.2017.06.010 PMC5667678

[18] H. Maric , G. Supic , L. Kandolf‐Sekulovic , V. Maric , Z. Mijuskovic , T. Radevic , M. Rajovic , Z. Magic , Melanoma Res 2019, 29, 596. 30950914 10.1097/CMR.0000000000000612

[19] E. R. Plimack , J. R. Desai , J. P. Issa , J. Jelinek , P. Sharma , L. M. Vence , R. L. Bassett , J. L. Ilagan , N. E. Papadopoulos , W. J. Hwu , Invest New Drugs 2014, 32, 969. 24875133 10.1007/s10637-014-0115-4 PMC4171200

[20] A. Ianevski , A. K. Giri , T. Aittokallio , Nucleic Acids Res. 2020, 48, W488. 32246720 10.1093/nar/gkaa216 PMC7319457

[21] P. Khongorzul , C. J. Ling , F. U. Khan , A. U. Ihsan , J. Zhang , Mol. Cancer Res. 2020, 18, 3. 31659006 10.1158/1541-7786.MCR-19-0582

[22] S. J. Patel , R. Jindal , K. R. King , A. W. Tilles , M. L. Yarmush , PLoS One 2011, 6, e19910. 21611132 10.1371/journal.pone.0019910 PMC3097212

[23] M. S. Carlino , J. Larkin , G. V. Long , Lancet 2021, 398, 1002. 34509219 10.1016/S0140-6736(21)01206-X

[24] C. Dumontet , J. M. Reichert , P. D. Senter , J. M. Lambert , A. Beck , Nat Rev Drug Discov 2023, 22, 641. 37308581 10.1038/s41573-023-00709-2

[25] L. Sigalotti , A. Covre , E. Fratta , G. Parisi , P. Sonego , F. Colizzi , S. Coral , S. Massarut , J. M. Kirkwood , M. Maio , J Transl Med 2012, 10, 185. 22950745 10.1186/1479-5876-10-185 PMC3539917

[26] T. Mori , S. J. O'Day , N. Umetani , S. R. Martinez , M. Kitago , K. Koyanagi , C. Kuo , T. L. Takeshima , R. Milford , H. J. Wang , V. D. Vu , S. L. Nguyen , D. S. Hoon , J. Clin. Oncol. 2005, 23, 9351. 16361635 10.1200/JCO.2005.02.9876 PMC2856438

[27] J. A. Gollob , C. J. Sciambi , B. L. Peterson , T. Richmond , M. Thoreson , K. Moran , H. K. Dressman , J. Jelinek , J. P. Issa , Clin. Cancer Res. 2006, 12, 4619. 16899610 10.1158/1078-0432.CCR-06-0883

[28] L. P. Martin , J. A. Konner , K. N. Moore , S. M. Seward , U. A. Matulonis , R. P. Perez , Y. Su , A. Berkenblit , R. Ruiz‐Soto , M. J. Birrer , Gynecol. Oncol. 2017, 147, 402. 28843653 10.1016/j.ygyno.2017.08.015 PMC6893864

[29] M. Campisi , S. K. Sundararaman , S. E. Shelton , E. H. Knelson , N. R. Mahadevan , R. Yoshida , T. Tani , E. Ivanova , I. Canadas , T. Osaki , S. W. L. Lee , T. Thai , S. Han , B. P. Piel , S. Gilhooley , C. P. Paweletz , V. Chiono , R. D. Kamm , S. Kitajima , D. A. Barbie , Front Immunol 2020, 11, 2090. 33013881 10.3389/fimmu.2020.02090 PMC7507350

[30] Y. Mao , W. Luo , L. Zhang , W. Wu , L. Yuan , H. Xu , J. Song , K. Fujiwara , J. I. Abe , S. A. LeMaire , X. L. Wang , Y. H. Shen , Arterioscler Thromb Vasc Biol 2017, 37, 920. 28302626 10.1161/ATVBAHA.117.309017 PMC5408305

[31] L. Guidi , G. Pellizzari , P. Tarantino , C. Valenza , G. Curigliano , Cancers (Basel) 2023, 15. 10.3390/cancers15041130. PMC9954056 36831473

[32] J. T. Coates , S. Sun , I. Leshchiner , N. Thimmiah , E. E. Martin , D. McLoughlin , B. P. Danysh , K. Slowik , R. A. Jacobs , K. Rhrissorrakrai , F. Utro , C. Levovitz , E. Denault , C. S. Walmsley , A. Kambadakone , J. R. Stone , S. J. Isakoff , L. Parida , D. Juric , G. Getz , A. Bardia , L. W. Ellisen , Cancer Discov 2021, 11, 2436. 34404686 10.1158/2159-8290.CD-21-0702 PMC8495771

[33] H. M. Haikala , T. Lopez , J. Kohler , P. O. Eser , M. Xu , Q. Zeng , T. J. Teceno , K. Ngo , Y. Zhao , E. V. Ivanova , A. A. Bertram , B. A. Leeper , E. S. Chambers , A. E. Adeni , L. J. Taus , M. Kuraguchi , P. T. Kirschmeier , C. Yu , Y. Shiose , Y. Kamai , Y. Qiu , C. P. Paweletz , P. C. Gokhale , P. A. Janne , Cancer Res. 2022, 82, 130. 34548332 10.1158/0008-5472.CAN-21-2426 PMC8732289

[34] O. Sordet , Q. A. Khan , Y. Pommier , Cell Cycle 2004, 3, 1095. 15326388

[35] C. Madeddu , C. Donisi , N. Liscia , E. Lai , M. Scartozzi , A. Maccio , Int. J. Mol. Sci. 2022, 23. 10.3390/ijms23126489. PMC9224267 35742933

[36] S. J. Seitter , R. M. Sherry , J. C. Yang , P. F. Robbins , M. L. Shindorf , A. R. Copeland , C. T. McGowan , M. Epstein , T. E. Shelton , M. M. Langhan , Z. Franco , D. N. Danforth , D. E. White , S. A. Rosenberg , S. L. Goff , Clin. Cancer Res. 2021, 27, 5289. 34413159 10.1158/1078-0432.CCR-21-1171 PMC8857302

[37] a) M. H. Saleh , L. Wang , M. S. Goldberg , Cancer Immunol. Immunother. 2016, 65, 787; 26646852 10.1007/s00262-015-1776-3 PMC11028536

[38] Y. Wu , Q. Li , Y. Kong , Z. Wang , C. Lei , J. Li , L. Ding , C. Wang , Y. Cheng , Y. Wei , Y. Song , Z. Yang , C. Tu , Y. Ding , T. Ying , Mol. Ther. 2022, 30, 2785. 35462042 10.1016/j.ymthe.2022.04.013 PMC9372316

[39] T. N. Iwata , C. Ishii , S. Ishida , Y. Ogitani , T. Wada , T. Agatsuma , Mol. Cancer Ther. 2018, 17, 1494. 29703841 10.1158/1535-7163.MCT-17-0749

[40] J. Huang , A. T. Agoston , P. Guo , M. A. Moses , Adv. Sci. (Weinh) 2020, 7, 2002852. 33344137 10.1002/advs.202002852 PMC7740099

[41] P. Guo , J. Huang , L. Wang , D. Jia , J. Yang , D. A. Dillon , D. Zurakowski , H. Mao , M. A. Moses , D. T. Auguste , Proc Natl Acad Sci U S A 2014, 111, 14710. 25267626 10.1073/pnas.1408556111 PMC4205631

[42] J. Yu , B. Qin , A. M. Moyer , S. Nowsheen , T. Liu , S. Qin , Y. Zhuang , D. Liu , S. W. Lu , K. R. Kalari , D. W. Visscher , J. A. Copland , S. A. McLaughlin , A. Moreno‐Aspitia , D. W. Northfelt , R. J. Gray , Z. Lou , V. J. Suman , R. Weinshilboum , J. C. Boughey , M. P. Goetz , L. Wang , J. Clin. Invest. 2018, 128, 2376. 29708513 10.1172/JCI97924 PMC5983332

[43] A. M. Newman , C. L. Liu , M. R. Green , A. J. Gentles , W. Feng , Y. Xu , C. D. Hoang , M. Diehn , A. A. Alizadeh , Nat. Methods 2015, 12, 453. 25822800 10.1038/nmeth.3337 PMC4739640

[44] Z. Chen , A. Huang , J. Sun , T. Jiang , F. X. Qin , A. Wu , Sci. Rep. 2017, 7, 40508. 28084418 10.1038/srep40508 PMC5233994

[45] R. Brinster , A. Kottgen , B. O. Tayo , M. Schumacher , P. Sekula , C. K. Consortium , BMC Bioinformatics 2018, 19, 78. 29499647 10.1186/s12859-018-2081-x PMC5833079

[46] J. Lee , H. R. Cho , G. D. Cha , H. Seo , S. Lee , C. K. Park , J. W. Kim , S. Qiao , L. Wang , D. Kang , T. Kang , T. Ichikawa , J. Kim , H. Lee , W. Lee , S. Kim , S. T. Lee , N. Lu , T. Hyeon , S. H. Choi , D. H. Kim , Nat. Commun. 2019, 10, 5205. 31729383 10.1038/s41467-019-13198-y PMC6858362

