# Supplementary material for: Epigenetic Reprogramming Potentiates ICAM1 Antibody Drug Conjugates in Preclinical Models of Melanoma
Source: Adv Sci (Weinh). 2024 Jun 14;11(30):2400203. doi: 10.1002/advs.202400203 (PMC11321650; doi:10.1002/advs.202400203)
Supplement: Supplementary file 1 — Supporting Information [file ADVS-11-2400203-s001.docx]

**
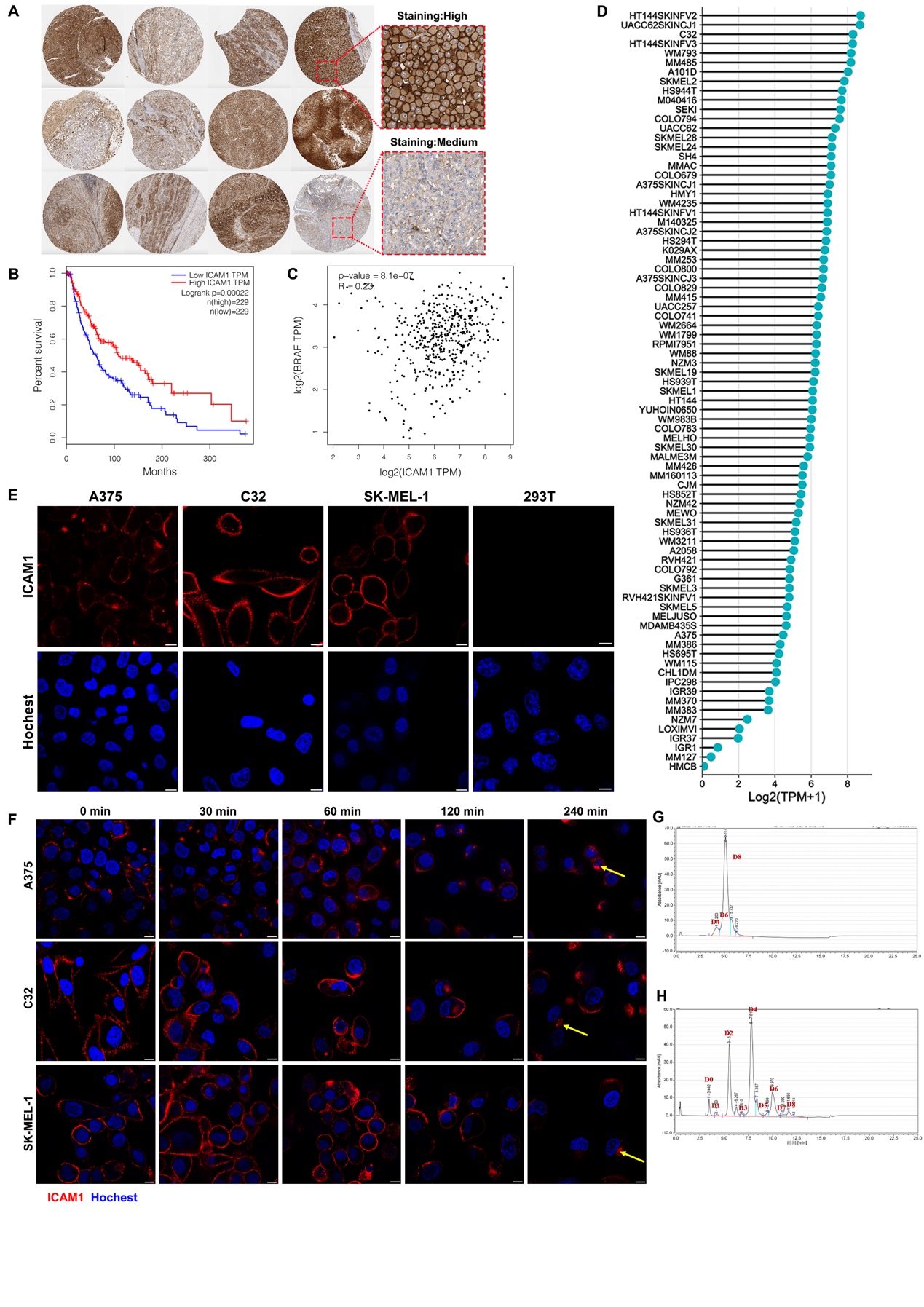
** **Figure S1.** ICAM1 was overexpressed in melanoma. (A) IHC of ICAM1 in 12 melanoma patients from Human Protein Atlas (<https://www.proteinatlas.org/>); 9/12 staining strong and 3/12 staining moderate; (B) Kaplan–Meier curve of overall survival of melanoma patients according to different ICAM1 expression levels in TCGA melanoma cohort (cutoff value: median ICAM1 expression level); (C) Pearson correlation analysis between ICAM1 mRNA expression and BRAF in TCGA melanoma cohort; (D) ICAM1 mRNA expression of 81 melanoma cell lines in the Cancer Cell Line Encyclopedia (CCLE); (E) IF staining of PE-ICAM1 antibody in melanoma cell line and 293T cell; (F) Representative images of IF staining of ICAM1 internalization in different time point (0min, 30min, 60min, 120min, 240min) in melanoma cell lines (A375; C32 and SK-MEL-1); yellow arrows indicate internalized ICAM1 antibody; (G) The DAR of I1-DXd was determined by HIC, displaying an average DAR of 8; (H) The DAR of I1-MMAE was determined by HIC, displaying an average DAR of 4.

**
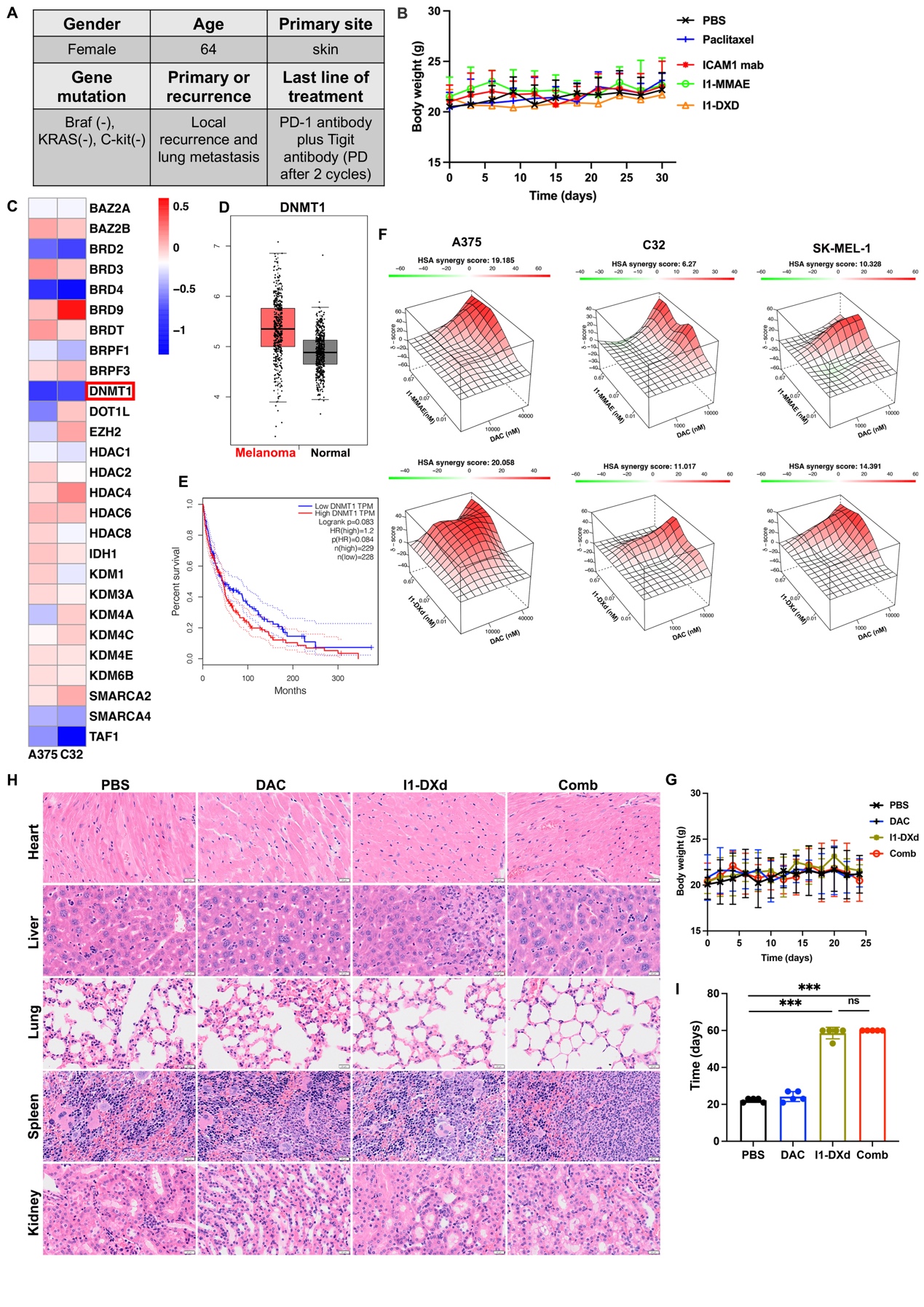
** **Figure S2.** (A) The clinical information of PDX; (B) Quantified mouse weight in different groups; (C) Essential score of epigenetic targets in A375 and C32 cell lines from the Cancer Dependency Map dataset (<https://depmap.org/portal/>); (D) Box plots compare DNMT1 mRNA levels via melanoma to normal tissue as obtained from GEPIA; (E) Kaplan–Meier analysis of disease-free survival of melanoma patients according to different ICAM1 levels in TCGA cohort; (F) Visualization of the calculated 3D synergy maps from SynergyFinder. The highest single agent (HSA) mode synergy score is presented as the average of all δ-scores across the dose-response matrix; (G) Quantified mouse weight in different groups; (H) HE staining of main organs, including heart, lung, liver, spleen and kidney in different groups. Scale bar equals to 20 μm; (I) Survival time of tumor-bearing mice in different groups; *** p <0.001.


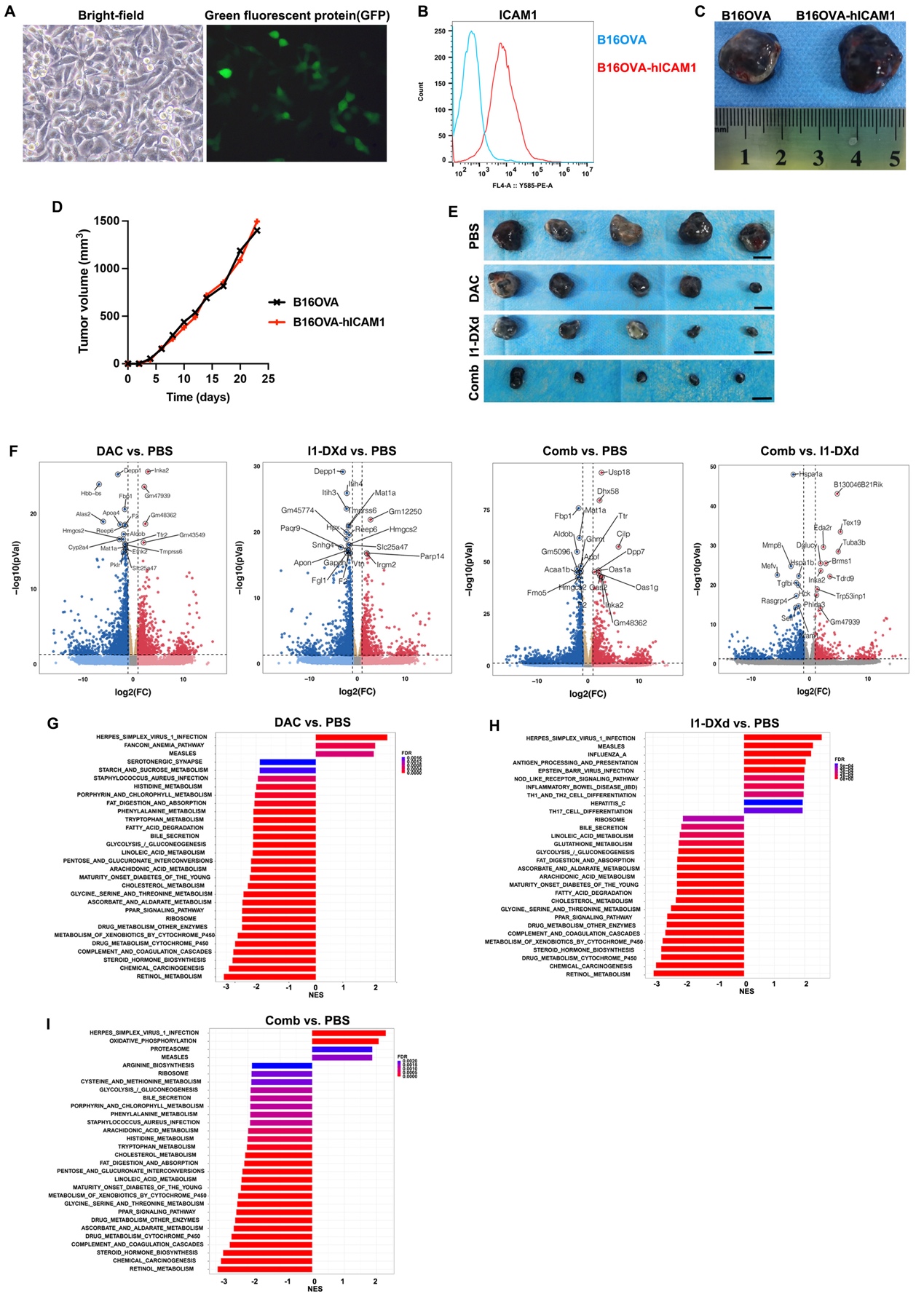
 **Figure S3.** (A) Construction of B16OVA-hICAM1 cell line; (B) B16OVA and B16OVA-hICAM1 surface expression of ICAM1 by flow cytometry (PE-labeled antibody); (C) Image of harvested B16OVA-hICAM1 tumor vs. parental B16OVA tumor; (D) Tumor progression curve in B16OVA-hICAM1 tumor or B16OVA tumor by tumor volume measurement by caliper; (E) Image of excised subcutaneous B16OVA-hICAM1 tumor from mice treated with PBS (sham), DAC, I1-DXd, or Combination group (n=5 per group). Scale bar equals to 1 cm; (F) Differential gene volcano map of different groups; (G) Top 30 GSEA enrichment analysis results of the KEGG pathways in DAC group vs. PBS group; (H) Top 30 GSEA enrichment analysis results of the KEGG pathways in I1-DXd group vs. PBS group; (I) Top 30 GSEA enrichment analysis results of the KEGG pathways in Comb group vs. PBS group.

**
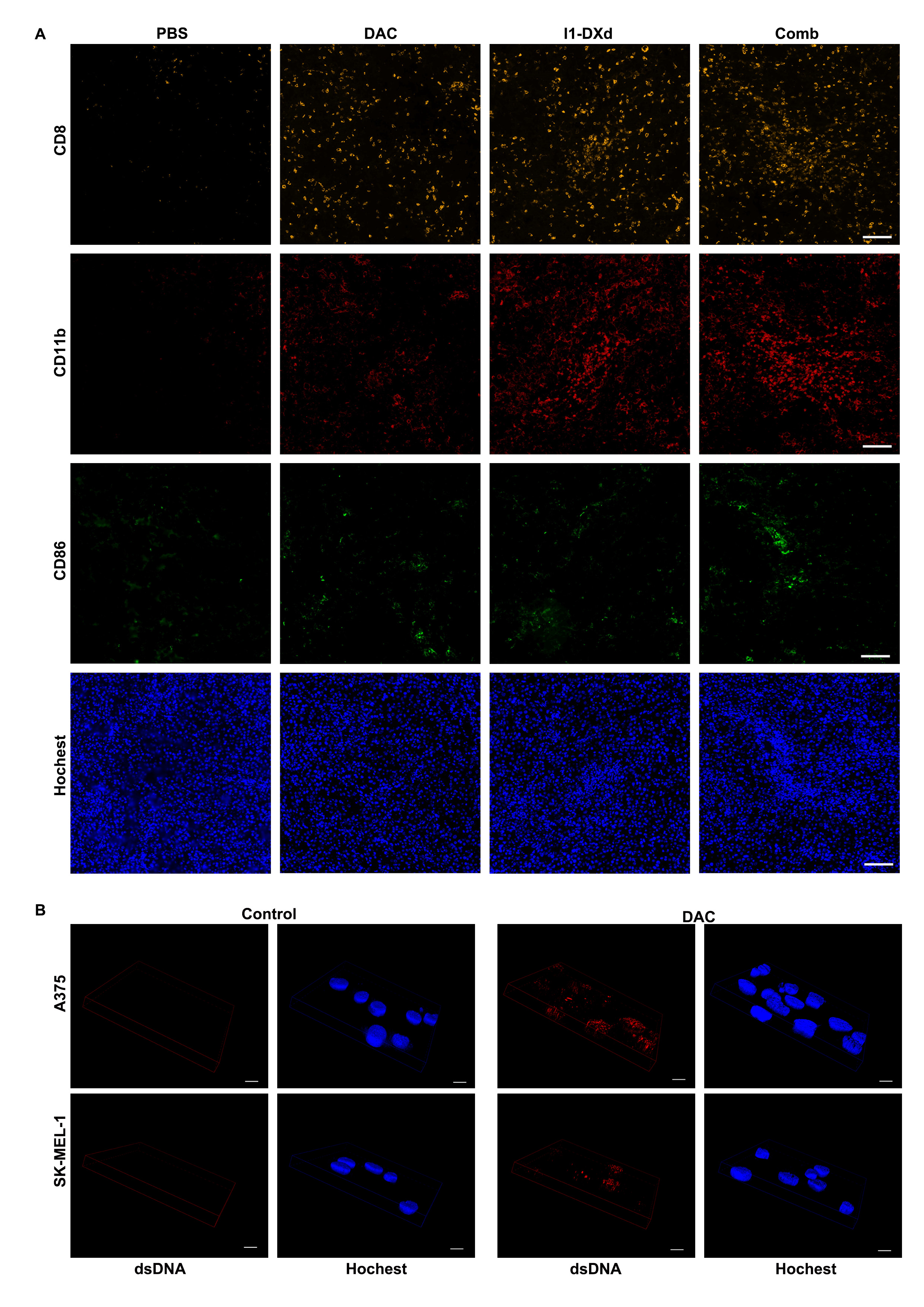
** **Figure S4.** (A) Representative multiplex immunofluorescence images of tumor samples in different groups for CD8 (pink), CD86 (green) and CD11b (red); (B) Representative images of IF staining of dsDNA in different groups.
